# Subregion preference in the long-range connectome of pyramidal neurons in the medial prefrontal cortex

**DOI:** 10.1186/s12915-024-01880-7

**Published:** 2024-04-29

**Authors:** Ayizuohere Tudi, Mei Yao, Feifang Tang, Jiandong Zhou, Anan Li, Hui Gong, Tao Jiang, Xiangning Li

**Affiliations:** 1 Britton Chance Center for Biomedical Photonics, Wuhan National Laboratory for Optoelectronics, MoE Key Laboratory for Biomedical Photonics, Huazhong University of Science and Technology, Wuhan, China; 2 HUST-Suzhou Institute for Brainsmatics, JITRI, Suzhou, China; 3 State Key Laboratory of Digital Medical Engineering, School of Biomedical Engineering, Hainan University, Haikou, China

**Keywords:** Medial prefrontal cortex, Pyramidal neurons, Whole-brain atlas, Input–output connectome, Topological connection

## Abstract

**Background:**

The medial prefrontal cortex (mPFC) is involved in complex functions containing multiple types of neurons in distinct subregions with preferential roles. The pyramidal neurons had wide-range projections to cortical and subcortical regions with subregional preferences. Using a combination of viral tracing and fluorescence micro-optical sectioning tomography (fMOST) in transgenic mice, we systematically dissected the whole-brain connectomes of intratelencephalic (IT) and pyramidal tract (PT) neurons in four mPFC subregions.

**Results:**

IT and PT neurons of the same subregion projected to different target areas while receiving inputs from similar upstream regions with quantitative differences. IT and PT neurons all project to the amygdala and basal forebrain, but their axons target different subregions. Compared to subregions in the prelimbic area (PL) which have more connections with sensorimotor-related regions, the infralimbic area (ILA) has stronger connections with limbic regions. The connection pattern of the mPFC subregions along the anterior–posterior axis showed a corresponding topological pattern with the isocortex and amygdala but an opposite orientation correspondence with the thalamus.

**Conclusions:**

By using transgenic mice and fMOST imaging, we obtained the subregional preference whole-brain connectomes of IT and pyramidal tract PT neurons in the mPFC four subregions. These results provide a comprehensive resource for directing research into the complex functions of the mPFC by offering anatomical dissections of the different subregions.

**Supplementary Information:**

The online version contains supplementary material available at 10.1186/s12915-024-01880-7.

## Background

It has been suggested that the medial prefrontal cortex (mPFC) differs from other cortical regions in its specific role in executive functions as well as its unique input and output connectivity patterns [1–3]. According to its cellular and chemical architecture, the mPFC is divided into the prelimbic area (PL), infralimbic area (ILA), and anterior cingulate area (ACA) [4]. Although both the PL and the ILA play important roles in working memory, decision making, emotion regulation, and social behavior [2, 5, 6], they have opposite functions in some aspects [7–9]. For example, PL is more related to the formation of fear memory, while ILA is more related to the extinction of fear memory [7, 10–12]. It has also been reported that PL activation produces anxiety-like behavior, whereas ILA activation has no effect [13, 14]. Pathological conditions such as chronic pain, addiction, and depression also have different impacts on the PL and ILA [15–18]. Notably, these functional studies focused mainly on specific sites in the mPFC, while the mPFC, especially the PL, spans a large area along the anterior–posterior (A-P) axis. Considering that most limbic areas, such as the insula cortex, hippocampus, and basolateral amygdalar nucleus (BLA), are heterogeneous along the A-P axis [19–21], it is rational to speculate that the mPFC may also contain heterogeneous structural and functional modules along the A-P axis.

The mPFC contains multiple projection neurons, including intratelencephalic (IT), pyramidal tract (PT), and corticothalamic (CT) neurons located in different layers, and they send long-range axons to target specific downstream areas. PT neurons project to the ipsilateral subcortical nuclei, while IT neurons mainly project to the bilateral cortex and striatum. Some studies have shown that there are functional differences among IT and PT neurons in the mPFC. For example, when conducting a delayed response task, working memory maintenance and time tracking are divided between IT and PT neurons in the mPFC, respectively [22]. In several mouse models of autism spectrum disorder, PT neurons in layer 5 are more easily affected than IT neurons [23, 24]. These functional differences may result from differences in input and output connectivity. Studies have shown that BLA inputs are stronger in PT neurons than in IT neurons in the L5 of the ILA, in contrast to thalamic inputs but similar to callosal inputs [25, 26]. Some studies have described the whole-brain inputs and outputs of cortical IT and PT neurons but have focused mainly on the sensorimotor cortex [27–29]. Additionally, the distribution patterns of long-range input circuits are similar for different types of GABAergic neurons in the same subregions [30, 31]. However, the whole-brain input–output connections of the various types of cortical projection neurons in the different subregions of the mPFC are still unclear.

Here, we provide a comprehensive whole-brain description of the input and output connections of deep-layer pyramidal neurons in the mPFC by using Cre driver mouse lines, virus tracing, and fluorescence micro-optical sectioning tomography imaging system (fMOST). In addition, we divided the mPFC into four parts: anterior, middle, posterior of the PL (aPL, mPL, pPL) and ILA along the A-P axis and compared the connectivity patterns of pyramidal neurons. We used Fezf2-CreER and Plxnd1-CreER transgenic mice to target PT and IT neurons, respectively, and found that different types of pyramidal neurons received inputs from similar brain regions with quantitative differences. Furthermore, our results revealed strikingly different input patterns between aPL and pPL, which indicated the structural and functional heterogeneity of PL along the A-P axis.

## Results

### Experimental strategy and whole-brain mapping of the input/output

**Fig. 1:**
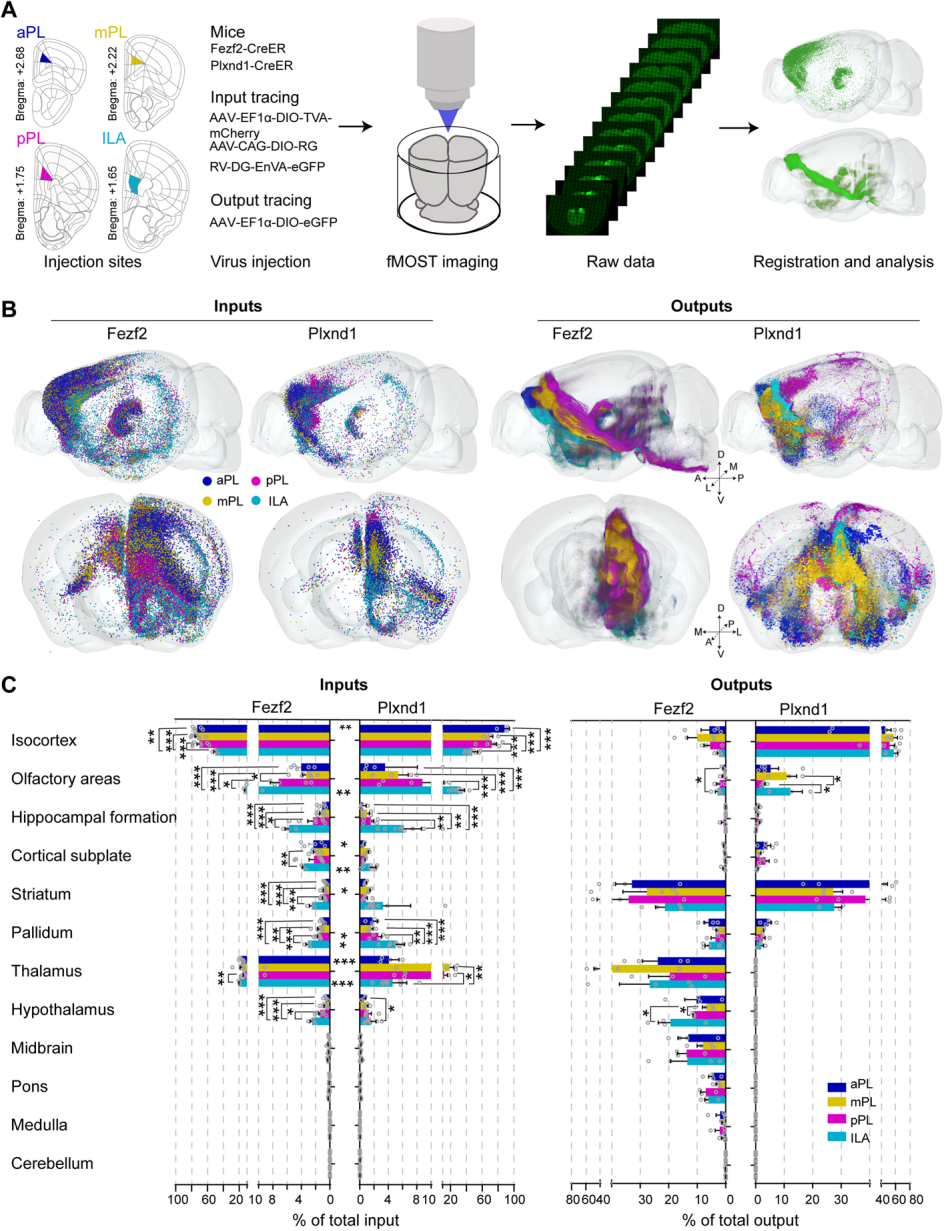
Experimental strategy and whole-brain overview of mPFC connectivity. **A** Schematic outlining viral tracing, whole-brain imaging, data processing, and analysis. Locations of four mPFC virus injection sites in this experiment: aPL (blue), mPL (yellow), pPL (purple), and ILA (cyan). **B** Three-dimensional representation of whole-brain inputs to and outputs from the four mPFC subregions in Fezf2 and Plxnd1 neurons. One dot represents one neuron. A, anterior; P, posterior; D, dorsal; V, ventral; M, medial; L, lateral. **C** Quantitative statistics of whole-brain input and output of the mPFC. The data are displayed as the average ± SEM. A two-sided Student’s *t*-test was used to generate *P* values. Significant differences are labeled as ***P < 0.001, **P < 0.01, *P < 0.05. Data from Fezf2 inputs: *n* = 5 (aPL), *n* = 7 (mPL and pPL), *n* = 3 (ILA); Plxnd1 inputs: *n* = 4 (aPL and mPL), *n* = 5 (pPL), *n* = 9 (ILA); Fezf2 outputs: *n* = 4 (aPL, mPL and ILA), *n* = 3 (pPL); Plxnd1 outputs: *n* = 5 (aPL), *n* = 3 (mPL, and ILA), *n* = 4 (pPL) animals

Considering that the PL is a large-span brain region along the A-P axis, in order to map the connectivity of the entire mPFC, four injection sites were selected: aPL, mPL, pPL, and ILA (corresponding to bregma 2.68 mm, 2.22 mm, 1.75 mm, and 1.65 mm) (Fig. 1A). Fezf2-CreER and Plxnd1-CreER transgenic mice, which express Cre recombinase in the isocortical PT and IT neurons, respectively, were used to obtain the whole-brain connectivity map of mPFC PT and IT neurons located in layers 5b and 6 and layer 5a (Additional file 1: Fig. S1A).

To study the whole-brain input, the AAV helper viruses AAV-DIO-RG and AAV-DIO-TVA-mCherry were injected into each target brain region (Fig. 1A). Tamoxifen (TM, 20 mg/kg) was used for induction on the third day, the RV virus RV-EnvA-△G-eGFP was injected at the same site 3 weeks later, and the animals were perfused after 1 week of RV virus expression. For the whole-brain output, the AAV-DIO-GFP virus was injected into the target brain area. TM was also used for induction on the third day, and the animals were perfused after three weeks of virus treatment. Then, the labeled mouse brains were plastically embedded in resin, and the embedded samples were continuously imaged by the fMOST system [32]. Next, the somas of the input neurons and the pixels of the output axon fibers were quantitatively calculated by Neuro-GPS at the whole-brain level [33], and the calculated results were registered to the standard Allen Brain Atlas with DeepMapi [34].

To verify the specificity of virus labeling, AAV-DIO-mCherry was injected into the mPFC of Fezf2-CreER;LSL-H2B-GFP mice, in which the soma of Fezf2 + neurons expressed GFP. Three weeks later, the mice were perfused, and 87 ± 3% of the mCherry-labeled neurons were found to coexpress GFP (Additional file 1: Fig. S1B), which demonstrated the high specificity of the virus. To ensure that we targeted the correct mPFC subregion via virus injection, we next assessed the spread of the starter cell populations for both RV and AAV tracing. For the RV experiments, starter cells were counted when they were double positive for RV-eGFP and TVA-mCherry (Additional file 1: Fig. S1C-D). For AAV tracing, starter cells were counted as eGFP-positive cell bodies (Additional file 1: Fig. S1E). We identified the distribution centers of starter cells and quantified the brain region distribution of the starter cells (Additional file 1: Fig. S2). The distribution centers of starter cells for the distinct mPFC subregions were broadly separated, and some overlapped for both RV and AAV tracings. In addition, the starter neurons were relatively restricted to the injection site, with some spreading to the adjacent frontal cortex brain region. The results showed that the connection features and variation in mPFC subregions could be basically reflected with viral labeling samples.

The mPFC showed extensive connectivity with many brain regions (Additional file 1: Fig. S4–S7, S9–S10). With the 3D representation of whole-brain inputs, we found that the input neurons of the mPFC were mainly distributed in the isocortex and thalamus, and the input neurons showed an outer-inner shell distribution, particularly in Fezf2 inputs, with the outer shell aPL enclosing the inner shell mPL, pPL, and ILA sequentially in the isocortex inputs but oppositely in thalamic inputs (Fig. 1B, Additional file 1: Fig. S3). To provide an overview of whole-brain connectivity, the input–output regions were divided into 12 larger regions (Fig. 1C). The results showed that in addition to the isocortex and thalamus, the mPFC also received input from the olfactory areas, hippocampal formation, cortical subplate, striatum, pallidum, and hypothalamus. There were statistically significant differences in the proportions of many input nuclei among the four subregions, whereas the main differences were found between PL and ILA (Fig. 1C and Additional file 1: Fig. S7). At the large brain region level, PL received more inputs from the isocortex, while ILA received more input from the hippocampal formation and olfactory area (Fig. 1C). ILA also received more inputs from the striatum and pallidum, which was consistent with our previous study [30]. More brain regions that differentially innervated Plxnd1 or Fezf2 neurons across the mPFC subregions are listed in Additional file 1: Fig. S11. Moreover, in the same subregion, the inputs to Plxnd1 neurons and Fezf2 neurons exhibited quantitatively different patterns (Fig. 1C and Additional file 1: Fig. S8).

With the 3D representation of whole-brain outputs, the four mPFC subregion projection pathways showed relatively spatially segregated patterns (Fig. 1B and Additional file 1: Fig. S3). For whole-brain output tracing, the Fezf2 neurons mainly projected to the ipsilateral subcortical brain areas, including the striatum, thalamus, hypothalamus, midbrain, and hindbrain, while the Plxnd1 neurons mainly projected to the ipsilateral and contralateral adjacent frontal cortex brain regions and striatum. For Plxnd1, the spatial distributions of the output fibers exhibited mirror symmetry across the midline of the brain, and the relative intensity was very similar across the contralateral and ipsilateral sides (Fig. 1B, C, Additional file 1: Fig. S3, S5, S6, and S10). Because of this symmetry, there was no special distinction between the ipsilateral and contralateral sides in the subsequent analysis. In addition, unlike the input patterns of the PL and ILA, we found that there was almost no difference in the output proportional distribution patterns among the four mPFC subregions (Fig. 1C), which indicates that different subregions of the mPFC may share similar output targets. Of course, there were also some brain regions in which Fezf2 or Plxnd1 neurons projected differently across the mPFC subregions (Additional file 1: Fig. S10, S11B-C).

### Interactions with the cortical regions

**Fig. 2:**
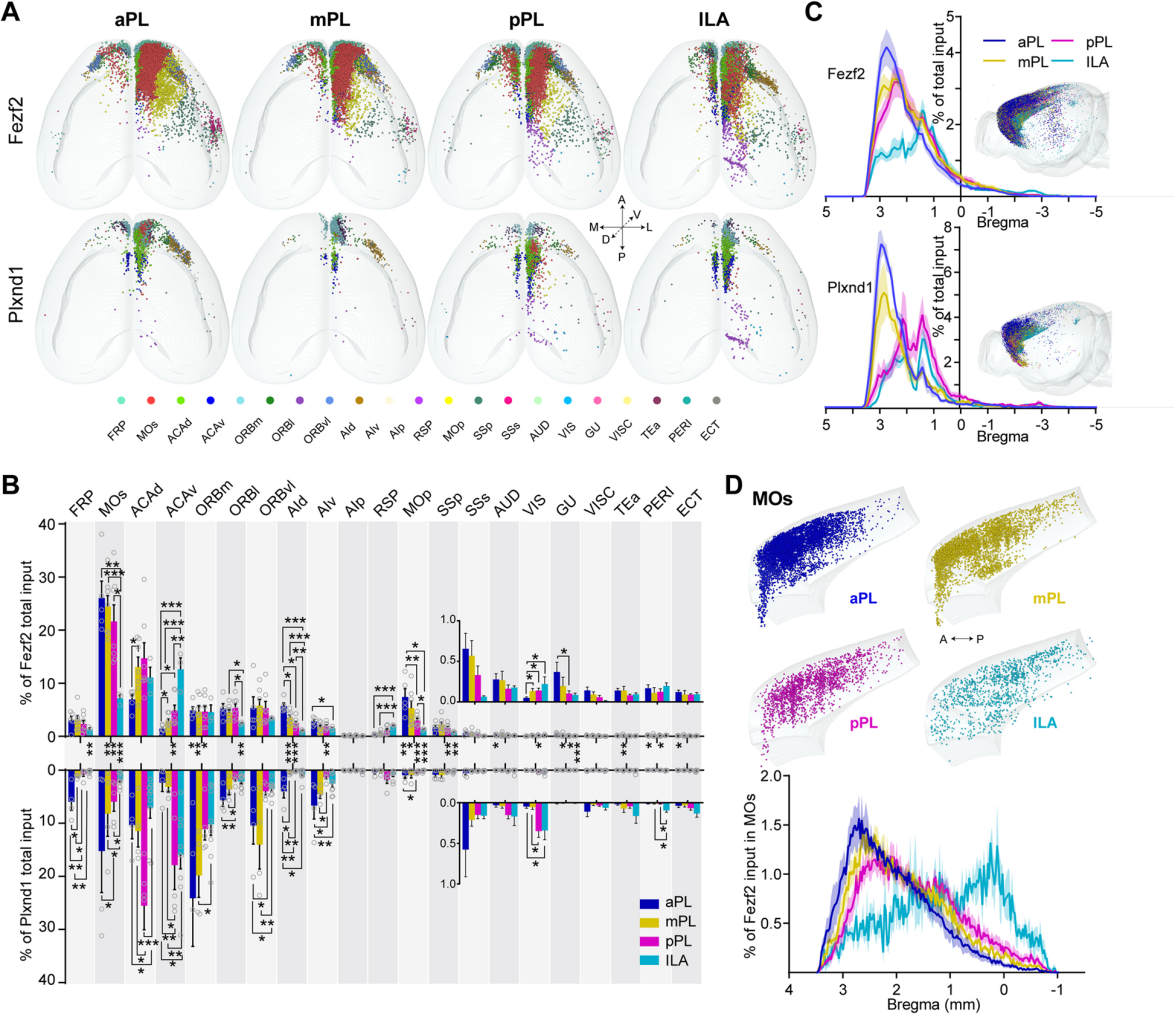
mPFC-isocortex connectivity. **A** Three-dimensional representation of the anatomical distribution of the input neurons from the isocortex to the Fezf2 and Plxnd1 neurons in mPFC subregions from representative samples. One dot represents one input neuron. The dots of different colors represent the input neurons of different isocortical brain regions. **B** Proportions of input in discrete isocortical regions according to the Allen Reference Atlas (ARA). Most of the labeled neurons were found within the prefrontal cortex subregions. A two-sided Student’s *t*-test was used to generate *P* values. Significant differences were labeled as ***P < 0.001, **P < 0.01, and *P < 0.05. **C** Input cell count density plots along the A-P axis, representing the isocortex distribution of all detected RV-eGFP-labeled neurons. The right inset shows a visual representation of the input neuron distribution in the isocortex. **D** Upper panel, lateral view of the distribution of input neurons from distinct mPFC subregions in the MOs. Lower panel, the input neuron density plot of the MOs along the A-P axis. The data in **B**–**D** are displayed as the average ± SEM, and the SEM is indicated by the shaded area in the density plot. Data from Fezf2 inputs: *n* = 5 (aPL), *n* = 7 (mPL and pPL), *n* = 3 (ILA); Plxnd1 inputs: *n* = 4 (aPL and mPL), *n* = 5 (pPL), *n* = 9 (ILA) animals. For more detailed brain region abbreviations, see Additional file 1: Table S1

The isocortex was the main input area of the mPFC, and the mPFC received almost all cortical brain region inputs (Figs. 1C and 2A, B). However, the input neurons were mainly distributed in the frontal cortex adjacent to the mPFC, including the frontal pole (FRP), the secondary motor cortex (MOs), the dorsal and ventral anterior cingulate areas (ACAd and ACAv), the orbital area (ORB), and the agranular insular cortex (AId and AIV, dorsal and ventral, respectively) (Fig. 2A, B). The cortical input patterns showed significant differences between the mPFC subregions (Fig. 2B). For example, the MOs, AId, and primary motor area (MOp) sent the most fibers to aPL and the least fibers to ILA, while the ACAv, retrosplenial area (RSP), and visual areas (VIS) sent the most inputs to the ILA and the least inputs to the aPL, and from aPL to mPL to pPL to ILA, the input proportions decreased or increased sequentially. (Fig. 2B). These results revealed that from the aPL to the ILA, the cortical input patterns showed a continuous shift along the A-P axis, which indicated that the PL may not be homogenous, as in the conventional view.

Additionally, even in the same subregion, different cell types showed quantitatively different inputs (Fig. 2B). For example, the MOs, MOp, and gustatory areas (GU) sent more fibers to Fezf2 than to Plxnd1. However, the opposite trend was observed for the medial part of the orbital area (ORBm). These results indicated that different cortical areas may form different functional motifs with Fezf2 and Plxnd1 in the mPFC to execute different functions.

In addition, cortical input neurons were distributed topologically between the mPFC subregions along the A-P axis (Fig. 2C). As the mPFC injection sites were shifted backward, the connecting centers in the isocortex shifted backward. It was particularly evident in the MOs that the ILA mainly received input from the posterior part of the MOs, while anterior MOs preferentially projected to the PL (Fig. 2D).

Next, we analyzed the layer distribution of the cortical input neurons. The cortical layer distribution of the inputs was region-dependent. Generally, cortical inputs mainly came from layers 2/3, followed by layer 5 (Additional file 1: Fig. S12B, S13A). The isocortical areas containing layer 4 are called the granular cortex, and the areas that do not contain layer 4 are called the agranular cortex (Additional file 1: Fig. S12A). Our results showed that more than 95% of the cortical inputs of the mPFC came from the agranular cortex (Additional file 1: Fig. S12C, S13B). Our previous study revealed that GABAergic neurons in the mPFC receive more input from layers 2/3 of the agranular cortex and layer 5 of the granular cortex [30]. For the inputs of pyramidal neurons in the present study, we found that the rule generally also holds true, especially for the Fezf2 input (Additional file 1: Fig. S12D), with some exceptions in several agranular cortices. For example, in the MOp, ACA, posterior part of the agranular insular area (AIp), lateral agranular part of the retrosplenial area (RSPagl), and perirhinal area (PERI) cortical regions, both layer 2/3 neurons and layer 5 neurons projected to Fezf2 neurons in the mPFC, while in the MOs, ventral part of the retrosplenial area (RSPv), and ectorhinal area (ECT) cortical regions, the input neurons were mainly located in layer 5 (Additional file 1: Fig. S12E). Based on these results, we came up with a detailed cortical-mPFC network model (Additional file 1: Fig. S12F). Overall, Fezf2 neurons in the mPFC received more inputs from layer 2/3 of the ORB and AI brain regions and layer 5 of other cortical regions. In addition, the laminar distribution pattern of cortical input neurons in each cortical area of Plxnd1 in the mPFC was largely different from that of neurons input to Fezf2 (Additional file 1: Fig. S13D), indicating that neurons located in different cortical layers may form different functional motifs with Fezf2 and Plxnd1 neurons in the mPFC.

### mPFC-thalamus connectivity

**Fig. 3:**
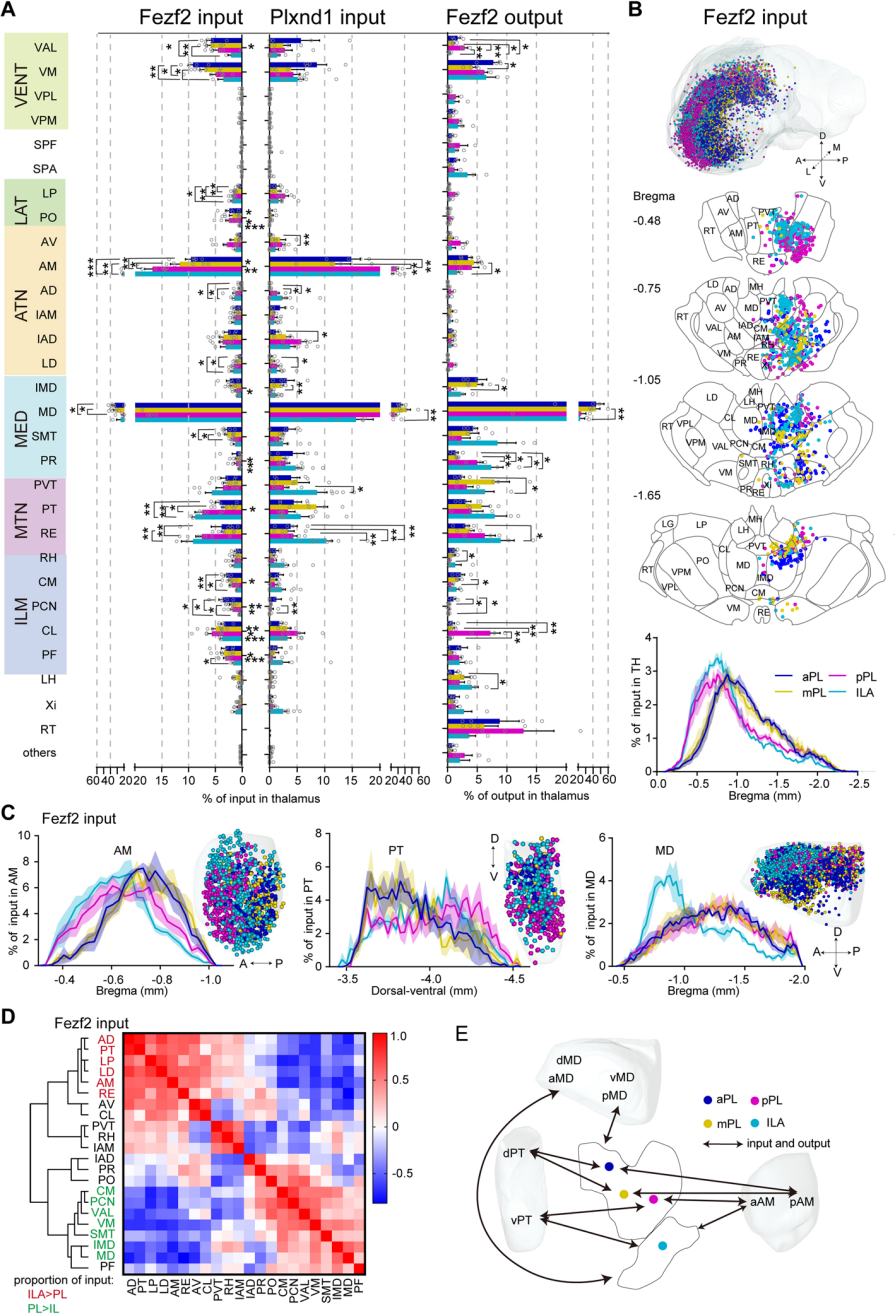
mPFC-thalamic connectivity. **A** Quantitative statistics of thalamic input and output of mPFC in Fezf2 and Plxnd1 neurons. A two-sided Student’s *t*-test was used to generate *P* values. Significant differences were labeled as ***P < 0.001, **P < 0.01, and *P < 0.05. **B** Three-dimensional illustration and coronal sections (thickness: 100 μm) depicting detected input neurons in the thalamus in Fezf2 from representative samples. One dot represents one input neuron, while different colors reflect inputs to different mPFC subregions. Lower panel, input cell density plot along the A-P axis, representing the distribution of all detected thalamic input neurons. **C** Comparison of inputs in the AM, PT, and MD regions to Fezf2 in the different mPFC subregions. Left panel, three-dimensional demonstration of input neurons in the mPFC from representative samples. Right, density plot of input neurons in the AM, PT, and MD along the A-P axis or dorsal–ventral axis. **D** Pearson’s correlation coefficient matrix and hierarchal clustering were used to investigate the clustering of thalamic nuclei according to their proportion of input to the Fezf2 neurons of the mPFC subregions in Fig. 3A. **E** Schematic of the connection patterns between thalamic nuclei (PT, MD, and AM) and mPFC subregions. a, anterior; p, posterior; d, dorsal; v, ventral. The data in **A**–**C** are displayed as the average ± SEM, and the SEM is indicated by the shaded area in the density plot. Data from Fezf2 inputs: *n* = 5 (aPL), *n* = 7 (mPL and pPL), *n* = 3 (ILA); Plxnd1 inputs: *n* = 4 (aPL and mPL), *n* = 5 (pPL), *n* = 9 (ILA); and Fezf2 outputs: *n* = 4 (aPL, mPL and ILA), *n* = 3 (pPL) animals. For more detailed brain region abbreviations, see Additional file 1: Table S1

We next analyzed the major subcortical connectivity component of the mPFC, the thalamus. Thalamocortical projections are thought to be essential relays and drivers of cortical activity in sensory areas and associative brain regions [35]. Cortico-thalamic feedback projections are sent from layer 6 and can shape thalamic cell activity via monosynaptic and disynaptic connections [36]. Whole thalamus analysis revealed that Fezf2 and Plxnd1 received inputs from similar thalamic nuclei (Fig. 3A). These thalamic nuclei formed reciprocal connections with Fezf2 in the mPFC, except for the reticular nucleus of the thalamus (RT) (Fig. 3A). The thalamic input neurons were mainly distributed in the anterior thalamic nuclei (ATN), medial dorsal thalamic nuclei (MED), midline thalamic nuclei (MTN), ventral thalamic nuclei (VENT), and intralaminar thalamic nuclei (ILM) ranging from bregma − 0.5 to − 2 (Fig. 3A, B). Comparing the mPFC subregions, ILA accepted more inputs from and sent more outputs to the MTN, including the paraventricular nucleus of the thalamus (PVT), parataenial nucleus (PT), and nucleus of reuniens (RE), while PL had more interconnections with the VENT and the ILM, including the ventral anterior-lateral complex of the thalamus (VAL), ventral medial nucleus of the thalamus (VM), central medial nucleus of the thalamus (CM), and paracentral nucleus of the thalamus (PCN). As expected, the brain region providing the largest proportion of input and output was the higher-order mediodorsal nucleus of the thalamus (MD) (Fig. 3A). PL/ILA had more efferent brain areas in the thalamus than the afferent, which may be overestimated because of passing fibers. Moreover, some thalamic nuclei also sent quantitatively different inputs to Fezf2 and Plxnd1 in the same subregion of the mPFC. For example, the VAL, PT, PCN, and central lateral nucleus of the thalamus (CL) sent more fibers to Fezf2 than to Plxnd1 (Fig. 3A). In addition, by comparing the differences between the inputs and outputs of mPFC Fezf2 neurons in thalamic areas, it was found that the input proportion of most nuclei with the input–output differences was larger than that of output, such as the anteromedial nucleus (AM), interanterodorsal nucleus of the thalamus (IAD), VAL, PCN, and CL (Additional file 1: Fig. S15).

In terms of spatial distribution, the inputs and outputs of the pPL and ILA were more distributed in the anterior part of the thalamus, which was most obvious in the thalamic input of Fezf2, and there was a similar trend in Plxnd1 input and Fezf2 output (Fig. 3B and Additional file 1: Fig. S14A–B). In the specific nucleus, pPL and ILA received more input from the anterior AM, the ventral PT, and the anterodorsal and lateral MD (Fig. 3C and Additional file 1: Fig. S14C, E). Correspondingly, the spatial distribution patterns of the outputs in the AM, PT, and MD were similar to those in the inputs (Additional file 1: Fig. S14D-E). Generally, the more backward the injection site in the mPFC was, the easier it was to connect with the anterodorsal thalamus. Based on our results, we came up with the spatial distribution of mPFC subregion-thalamus network models (Fig. 3E).

To further understand how the mPFC cortico-thalamic connection was organized, we performed hierarchical clustering based on the input from Fezf2 (Fig. 3D). Clustering analysis identified four major groups of nuclei across the thalamus. Thalamic nuclei in the two major groups displayed covariation in their input proportional distribution from PL to ILA. Thalamic nuclei, including the anterodorsal nucleus (AD), PT, lateral dorsal nucleus of the thalamus (LD), AM, lateral posterior nucleus of the thalamus (LP), and RE, which received inputs mainly from visual-related areas in the posterior cortex [37], were clustered into one group, and they sent more inputs to the ILA than to the PL. Thalamic nuclei, including the intermediodorsal nucleus of the thalamus (IMD), MD, CM, PCN, VAL, VM, and submedial nucleus of the thalamus (SMT), were clustered into one group, and they preferentially sent more inputs to the PL than to the ILA. Similar hierarchical clustering patterns were also detected for Plxnd1 input and Fezf2 output in the thalamus (Additional file 1: Fig. S14F–G).

### mPFC-basal forebrain, amygdala, and hypothalamus connectivity

The basal forebrain is an important area that regulates the functions of the mPFC. Decreased acetylcholine release in the mPFC from the basal forebrain severely disrupted attention performance and short-term memory [38, 39]. However, how different types of pyramidal neurons connect with the basal forebrain has not been comprehensively compared.

**Fig. 4:**
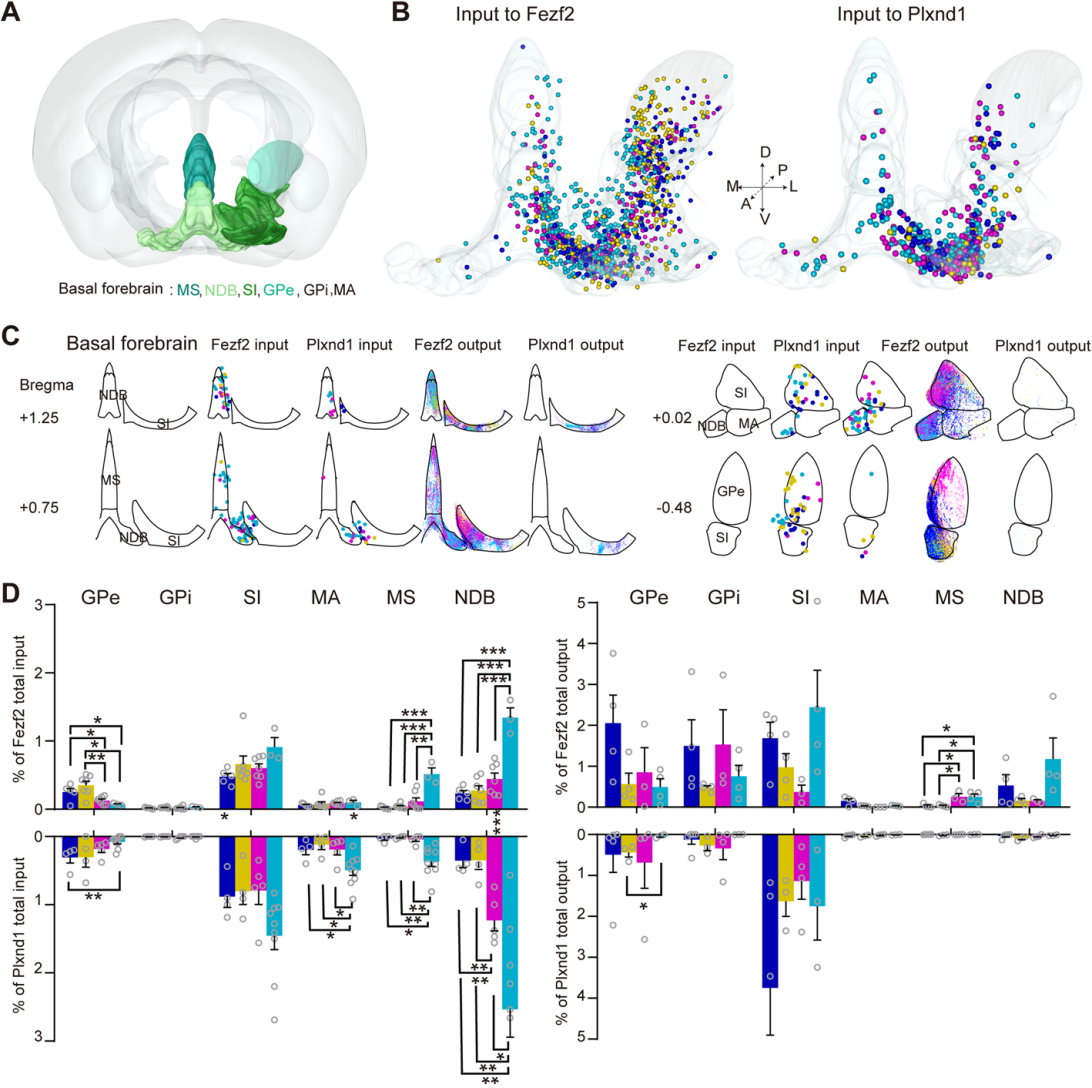
mPFC-basal forebrain connectivity. **A** The main brain areas contained in the basal forebrain. **B** Three-dimensional illustration of the basal forebrain inputs to the mPFC subregions in Fezf2 and Plxnd1 neurons. One dot represents one input neuron. **C** Schematic coronal sections (thickness: 100 μm) depicting detected basal forebrain inputs to and outputs from the mPFC subregions in Fezf2 and Plxnd1 neurons. **D** Quantitative statistics of the anatomical distribution of the input and output from the basal forebrain. The data are displayed as the average ± SEM. A two-sided Student’s *t*-test was used to generate *P* values. Significant differences were labeled as ***P < 0.001, **P < 0.01, and *P < 0.05. Data from Fezf2 inputs: *n* = 5 (aPL), *n* = 7 (mPL and pPL), *n* = 3 (ILA); Plxnd1 inputs: *n* = 4 (aPL and mPL), *n* = 5 (pPL), *n* = 9 (ILA) animals

Here, we compared the detailed mPFC-basal forebrain connectivity based on our anterograde and retrograde tracing results. Different pyramidal neurons in the mPFC received inputs from similar basal forebrain nuclei, including the medial septal complex (MS), diagonal band nucleus (NDB), substantia innominata (SI), globus pallidus, internal and external segment (GPi, GPe), and magnocellular nucleus (MA) (Fig. 4A–D). Interestingly, we found that some nuclei from the basal forebrain sent biased inputs to different subregions of the mPFC. For example, aPL and mPL received more inputs from GPe and sent more projections to GPe compared to pPL and ILA. Compared to PL, ILA had more connections with the MS and NDB, including more inputs and more projections (Fig. 4D). As reported in previous studies, GPe is more involved in motor control and modulation [40], while the MS and NDB play a role in attention, learning, and memory [41], which means that the PL and ILA may play different roles through the above preferential connections with the basal forebrain.

For different types of pyramidal neurons, the projection patterns of Fezf2 and Plxnd1 in the basal forebrain were different, while the input circuits were similar. Fezf2 neurons projected to the MS and NDB, but Plxnd1 rarely projected to them. Fezf2 and Plxnd1 neurons rarely received input from GPi, whereas they sent a large number of outputs to GPi, especially Fezf2 (Fig. 4C, D).

Similar to the basal forebrain, the amygdala and the mPFC were also reciprocally connected. The amygdala can be divided into the cortical pallial amygdala (superficial or cortical-like amygdala) and the deep pallial amygdala, and we found that the inputs to the Fezf2 and Plxnd1 neurons mainly came from the deep pallial amygdala (Additional file 1: Fig. S16). The output of Plxnd1 was also mainly concentrated in the deep pallial amygdala, especially in the BLA and basomedial amygdalar nucleus (BMA). Interestingly, the projections of Fezf2 were more restricted, and there were significant differences between Fezf2 projections from PL and ILA that aPL mainly projected to the anterior part of the BLA (BLAa), while ILA mainly projected to the cortical amygdala area (COA), BMA, and medial amygdalar nucleus (MEA).

Furthermore, we investigated the connections between the BLAa and different subregions of the mPFC, which showed spatial topological correspondence (Additional file 1: Fig. S17). In terms of the Fezf2 and Plxnd1 outputs, aPL and mPL preferentially projected to the anterolateral of the BLAa, while pPL and ILA projected more to the posteromedial BLAa. A similar distribution pattern was also found for the Fezf2 input, especially along the A-P axis.

The hypothalamus and mPFC were also reciprocally connected, and the input and output proportions of ILA in the hypothalamus were always greater than those in PL (Fig. 1C). The mPFC was mainly bidirectionally connected to the lateral preoptic area (LPO), lateral hypothalamic area (LHA), zona incerta (ZI), and posterior hypothalamic area (PH). In addition, the mPFC hardly accepted inputs from brain regions such as the parasubthalamic nucleus (PSTN), subthalamic nucleus (STN), and fields of Forel (FF), but Fezf2 neurons projected a large number to them. The hypothalamic input areas of Fezf2 and Plxnd1 neurons were very similar, and there were almost no significant differences between them (Additional file 1: Fig. S18).

### mPFC-hippocampal formation connectivity

**Fig. 5:**
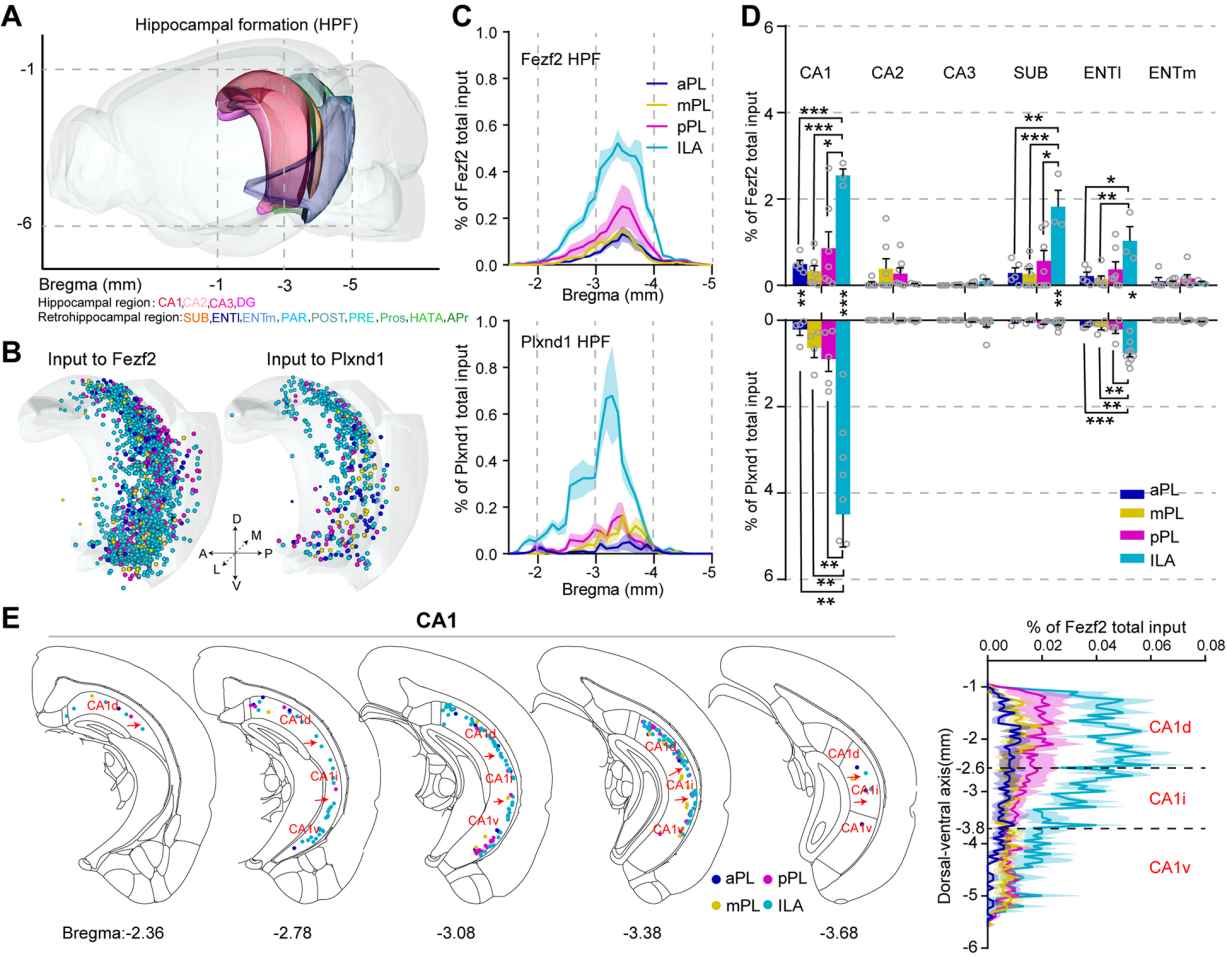
mPFC-hippocampus connectivity. **A** Schematic of the regions contained in the hippocampal region along the A-P axis according to the ARA. For more detailed abbreviations, see Additional file 1: Table S1. **B** Three-dimensional illustration of the hippocampal inputs to the mPFC subregions in Fezf2 and Plxnd1 neurons. **C** Input cell count density plots of Fezf2 and Plxnd1 neurons along the A-P axis. **D** Quantitative statistics of hippocampal inputs to the mPFC subregions in Fezf2 and Plxnd1 neurons. A two-sided Student’s *t*-test was used to generate *P* values. The data are displayed as the average ± SEM. Significant differences were labeled as ***P < 0.001, **P < 0.01, and *P < 0.05. **E** Schematic coronal sections (thickness: 100 μm) depicting the input neurons to Fezf2 in CA1. Right panel, normalized density plot of input neurons in CA1 in different mPFC subregions along the dorsal–ventral axis. The data in **C**–**E** are displayed as the average ± SEM, and the SEM is indicated by the shaded area in the density plot. Data from Fezf2 inputs: *n* = 5 (aPL), *n* = 7 (mPL and pPL), *n* = 3 (ILA); Plxnd1 inputs: *n* = 4 (aPL and mPL), *n* = 5 (pPL), *n* = 9 (ILA) animals

The mPFC received input from both the hippocampal region and the retrohippocampal region, and the input neurons were mainly distributed in the CA1, subiculum (SUB), and lateral part of the entorhinal area (ENTl) brain regions (Fig. 5A–D). Interestingly, we found that ILA received significantly more inputs from the hippocampal formation (HPF) than PL in both cell types (Fig. 5B–D), which is consistent with previous findings [42]. Plxnd1 in the ILA received more inputs from CA1 and ENTl, while Fezf2 in the ILA received more inputs from CA1, ENTl, and SUB (Fig. 5D). CA1 is divided into three major domains: dorsal (CA1d), intermediate (CA1i), and ventral (CA1v), and CA1d is primarily involved in the cognitive process of learning and memory associated with navigation, exploration, and locomotion, whereas CA1v is a part of the temporal lobe associated with motivational and emotional behavior [20]. We found that all three parts of CA1sent projections to both mPFC cell types, and compared with those in the CA1v, more input neurons were detected in the CA1d (Fig. 5E).

### Subregion preference of projections in other regions

**Fig. 6:**
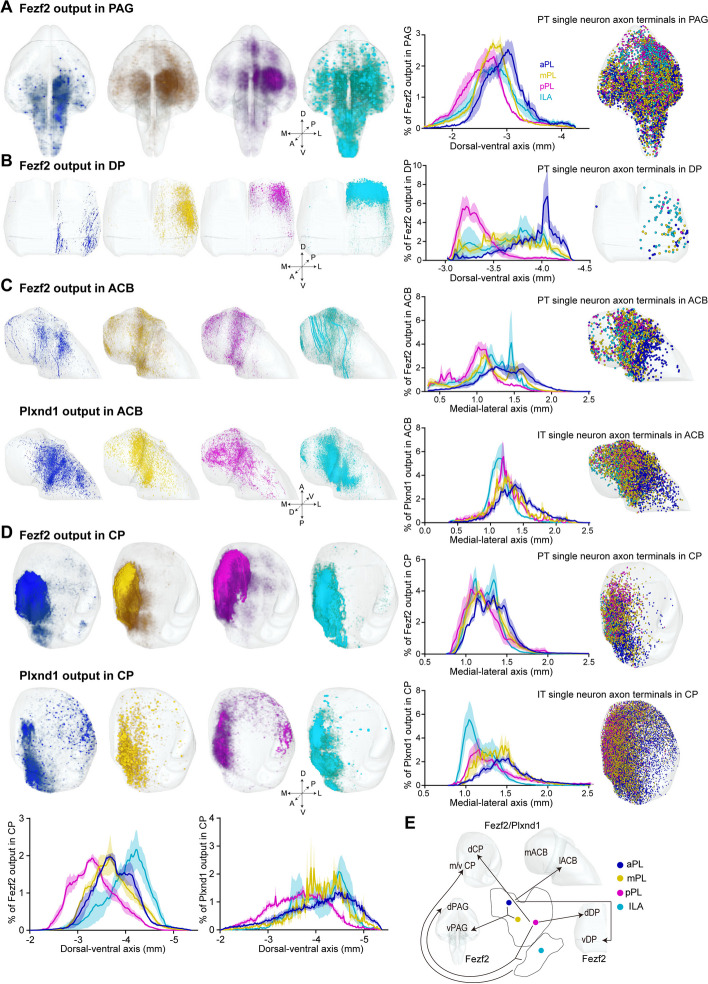
Several mPFC output nuclei showing differences in spatial distribution. **A**–**D** Left column, three-dimensional representation of the output axon fibers in the PAG (**A**), DP (**B**), ACB (**C**), and CP (**D**) from representative Fezf2 or Plxnd1 output samples. The intermediate column shows the density plot of outputs along the dorsal–ventral axis or medial–lateral axis. The right column shows the axon terminal distribution of single neurons in these brain regions, and one dot represents one axon terminal. **E** Schematic of the connection patterns in the PAG, DP, ACB, and CP regions among the mPFC subregions. Not all the connection features were summarized in the figure, only the more significant connection features. Both Fezf2 and Plxnd1 neurons projected to the CP and ACB, and the differences in their spatial distributions among mPFC subregions showed similar characteristics; therefore, they were combined. Fezf2 neurons projected to PAG and DP with the spatial distribution difference when Plxnd1 did not project to PAG, so the projection pattern of the mPFC to the PAG and DP referred to the projection pattern of Fezf2 neurons. d, dorsal; v, ventral; m, medial; l, lateral. The density plot data in **A**–**D** are displayed as the average ± SEM, and the SEM is indicated by the shaded area. Data from Fezf2 outputs: *n* = 4 (aPL, mPL and ILA), *n* = 3 (pPL) animals. SEM is indicated by the shaded area

Additionally, we also found some differences in the spatial distribution of other output nuclei between the mPFC subregions. We observed that aPL and mPL projected more to the ventral side of the periaqueductal gray (PAG), while pPL and ILA preferentially projected to the dorsal side of the PAG (Fig. 6A). The PAG is divided into several anatomical subgroups: dorsomedial, dorsolateral, lateral, and ventrolateral [43]. It has been proposed that the mPFC-dorsolateral projection contributes to defense responses such as unpleasant and compulsive behavioral reactions [44], and the mPFC-ventrolateral projection contributes more to pain regulation [45]. Therefore, it is speculated that pPL and ILA play more significant roles in the defense response. In addition, aPL preferentially projected to the ventral side of the dorsal peduncular area (DP), while pPL did the dorsal side for Fezf2 neurons. And aPL tended to preferentially project to the lateral side of the nucleus accumbens (ACB) while pPL did the medial side in both the Fezf2 and Plxnd1 cell types (Fig. 6B, C). Both the Fezf2 and Plxnd1 neurons projected to the caudoputamen (CP), and the projection distribution of them in CP is very similar, the main difference is that the projection range of Plxnd1 neurons is relatively wide, while that of Fezf2 neurons is relatively more concentrated. And pPL preferentially projected to the dorsal side of the CP while ILA preferentially projected to the ventromedial side of the CP (Fig. 6D). To further verify the topological distribution pattern of these output nuclei, we extracted the single neuron axonal terminals, which refer to the terminal boutons at the end of the axon segments [46] (Additional file 1: Fig. S19) of the reconstructed PT and IT single neurons from layer 5 of the four mPFC subregions (the right column in Fig. 6), and we found that the extracted terminals had a spatial distribution similar to that of the population output axon fibers. Single neurons were extracted from the website https://mouse.braindatacenter.cn/. The spatial distribution of these nuclei showed that the more forward the injection site in the mPFC was, the more projections there were to the ventrolateral side of the output nuclei.

### Whole-brain connectivity logic of the mPFC

**Fig. 7:**
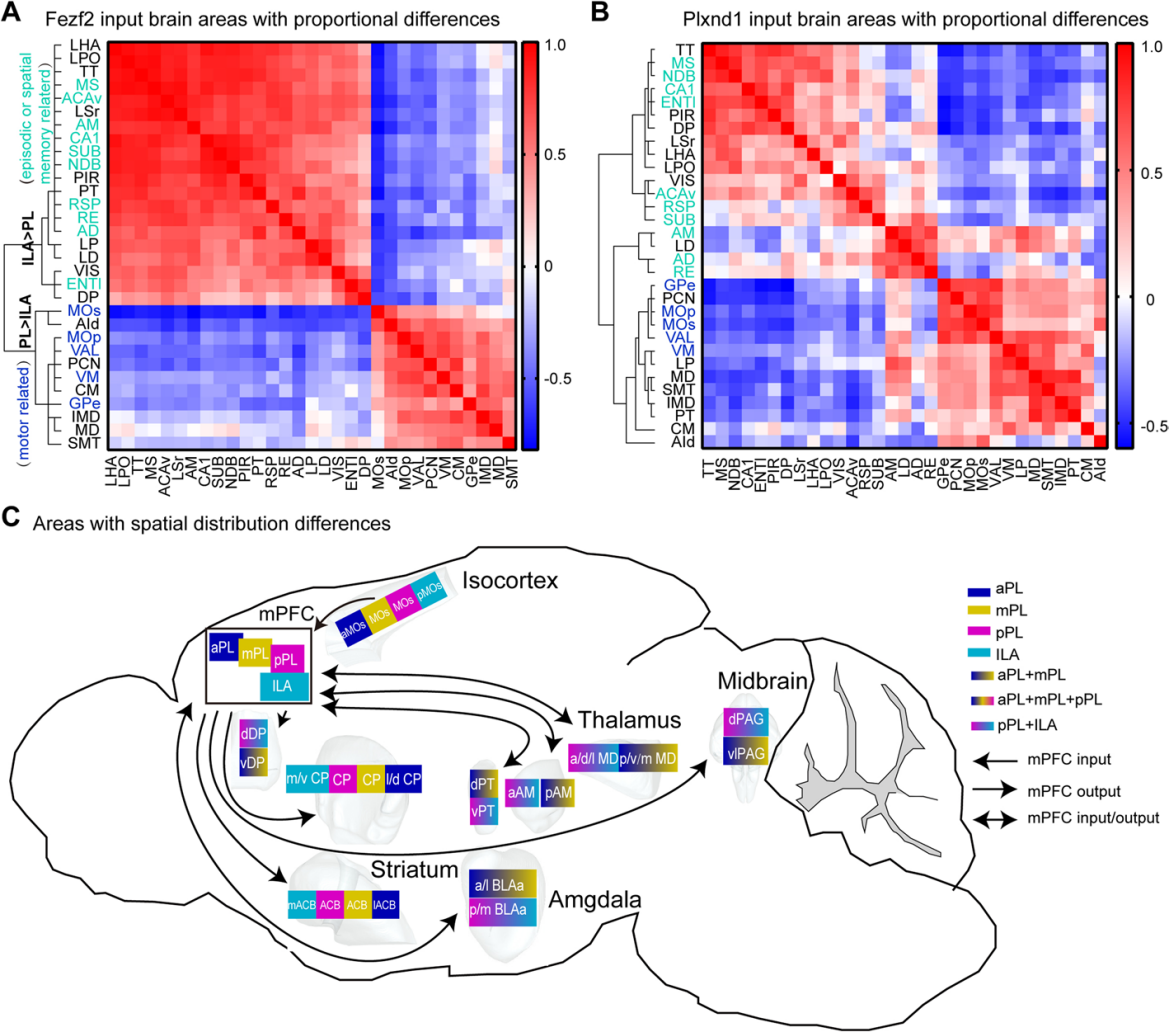
Connectivity characteristics of the mPFC subregions. **A**, **B** Spearman correlation matrix and hierarchal clustering showing the clustering of Fezf2 (**A**) and Plxnd1 (**B**) input brain areas with proportional differences based on their proportion of input to the mPFC subregions displayed in Additional file 1: Fig. S11A. Red, positive correlation; blue, negative correlation. **C** Whole-brain schematic of the areas with spatial distribution differences between the different mPFC subregions. a, anterior; p, posterior; d, dorsal; v, ventral; m, medial; l, lateral. For detailed abbreviations, see Additional file 1: Table S1

Throughout our analysis, distinctions emerged between the different subregions of the mPFC we targeted. To better illustrate proportional differences in whole-brain input, we performed correlation and hierarchical cluster analysis based on the proportions of differentially expressed nuclei from the Fezf2 and Plxnd1 inputs (Fig. 7A, B). We found that, compared with PL, nuclei preferentially projecting to ILA were reunited into a large category, including the MS, ACAv, AM, CA1, SUB, NDB, RSP, RE, AD, and ENTl, and many of these nuclei are related to episodic memory or spatial memory [47–49]. The nuclei projecting more to the PL were also grouped, and many of them are motor-related, such as the MOs, MOp, VAL, VM, and GPe. These results suggested that PL and ILA may be involved in different functions.

In addition, there were some differences in the distribution of input neurons among PL subregions, especially between aPL/mPL and pPL, and there was almost no difference between aPL and mPL (Additional file 1: Fig. S20A–B). Similarly, the input nuclei with different proportions among PL subregions were analyzed by hierarchical cluster analysis, and they were divided into two categories for Fezf2 input, in which from aPL to pPL, the proportion of input nuclei changed continuously (Additional file 1: Fig. S20C). For the first category, the proportion decreased continuously from aPL to mPL to pPL, including the MD, MOp, VM, and other nuclei, while the proportion increased continuously in the second category, including the ACAv, Taenia tecta (TT), piriform area (PIR), and other nuclei. The Plxnd1 input was slightly different and was divided into three main categories, two of which were similar to the Fezf2 input, and the third type included the brain regions with the most mPL input, such as the MD, PT, and IMD (Additional file 1: Fig. S20D).

We also summarized the nuclei showing differences in spatial distribution (Fig. 7C). Among them, pPL and ILA received more input from and sent more outputs to the anterior AM, ventral PT, anterodorsal and lateral MD, and posteromedial BLAa. Moreover, aPL and mPL projected to the ventral side of the PAG, and aPL preferentially projected to the ventral side of the DP nucleus, the lateral side of the ACB, and the ventrolateral side of the CP nucleus.

To test whether the observations described above represent meaningful differences, we correlated all the input and output tracings to each other in an unbiased manner. By unbiased clustering of the input and output circuits of the four mPFC subregions, we found that the outputs of both types of neurons in the four subregions showed high correlation coefficients, which indicated that the outputs of the four subregions were similar to one another. However, the inputs to the two types of neurons at the four subregions showed low correlation coefficients, especially in the inputs of Plxnd1, which indicated that the input patterns of the PL and the ILA clearly differed (Additional file 1: Fig. S21A). For the correlation matrix of individual input or output samples, the outputs of different subregions of PL and ILA formed one cluster, except for the two samples of ILA in Fezf2 and one sample of aPL in Plxnd1, indicating that the difference between the outputs of PL and ILA was very small (Additional file 1: Fig. S21B). However, the difference in the input brain regions was greater than that in the output brain regions. For the input matrix, aPL/mPL and ILA were included in different clusters, while pPL formed clusters with either aPL/mPL or ILA, which indicated that the input connectivity patterns of the pPL showed some level of similarity to the ILA.

## Discussion

Many brain areas, such as the hippocampus [20], BLA [50], PIR [51, 52], and striatum [53], have shown their structural and functional differences along the A-P axis. The mPFC, especially PL, spans more than 1 mm along the A-P axis; however, the differences between the mPFC subregions along the A-P axis or the dorsal–ventral axis have not been systematically compared.

By performing input and output tracing, we found that the output patterns of pyramidal neurons in four subregions of the mPFC were similar, while the input patterns showed specific differences. Some brain regions, including the brain regions of the isocortex, thalamus, basal forebrain, and hippocampus, sent quantitatively different inputs to different subregions of the mPFC (Figs. 1, 2, 3, 4, and 5). In addition, the input patterns showed a continuous change from aPL to pPL. The functions of pPL have been well studied [54, 55]; however, the functions of aPL are still largely unknown. Considering that the connectivity patterns of the pPL showed some level of similarity to the ILA and the function differences of the PL and ILA have been well characterized [10], it is rational to predict that the functions of the aPL and pPL could also differ in some aspects.

We also found that, compared with ILA, PL had more connections with the sensorimotor cortex and motor-related brain areas including the VAL, VM, and GPe. In contrast, ILA had rather extensive connections with limbic association cortices and subcortical areas, such as the ACAv, RSP, MS, NDB, CA1, SUB, ENTl, AM, AD, and RE. These connection patterns suggest that PL may belong to the dorsomedial PFC, while ILA may belong to the ventromedial PFC [56]. These results provided a quantitative investigation of the proposed dorsal–ventral dissociation of the rodent mPFC [57].

Additionally, we found that several input or output brain areas had topological connections with the four mPFC subregions. For example, aPL and mPL received more inputs from and sent more outputs to the anterolateral of the BLAa, while pPL and ILA reciprocally connected more with the posteromedial BLAa. It has been reported that activating ILA-pBLA inputs strengthens reward generalization and suppresses anxiety- and depression-like behaviors [58]. Moreover, photostimulation of pBLA-vCA1 inputs has an anxiolytic effect on mice, while stimulating aBLA-vCA1 inputs induces anxiety-like behavior [50]. Therefore, it is rational to predict that activating aPL–aBLA inputs may induce anxiety-like behavior. In addition to the BLAa, there were some other brain regions with topological distributions along the mPFC A-P or dorsal–ventral axis, including the AM, PT, MD, PVT, CP, ACB, and PAG, and we speculate that these regions may contain smaller subregions involving opposite brain functions or different aspects of the same function.

We also compared the differences in the input patterns of different pyramidal neurons in the same subregions. Some cortical regions, such as the ORBm and ACAv, tended to send more inputs to Plxnd1 in the mPFC, while the MOs, MOp, AId, and CL tended to send more inputs to Fezf2. The biased thalamic input to different types of excitatory neurons in the motor cortex has been described previously [59]. These biased inputs may be due to differences in axon innervation in different layers of the mPFC since the Plxnd1 and Fezf2 positive neurons occupy different layers of the cortex. The functional differences of these biased inputs need to be further investigated in future studies.

## Conclusions

In summary, we performed a detailed analysis of the distributions of the input and output of two major excitatory neurons in different subregions of the mPFC, obtained the subregional preference whole-brain connectome in four subregions, and identified continuous changes in input patterns from aPL to ILA, which can facilitate functional studies of the different subregions of the mPFC.

## Methods

### Animals

For the RV and AAV tracing, adult (2–6 months old) male and female Plxnd1-2A-CreER (strain no. 036294) and Fezf2-2A-CreER (strain no. 036296) [27] (a gift from Josh Huang’s laboratory, Cold Spring Harbor) mice were used. The C57BL/6 J mice used in these experiments were purchased from Beijing Vital River (Beijing). Ai3 (strain no. 007903) reporter mice were purchased from Jackson Laboratory, and the LSL-H2B-GFP (strain no. 036761) reporter mice were a gift from Josh Huang’s laboratory. All mice were housed in an environment with a 12-h light/dark cycle at 22 ± 1 °C and food and water were available ad libitum. This study was approved by the Institutional Animal Experimentation Ethics Committee of Huazhong University of Science and Technology, and all animal experiments were conducted following relevant guidelines.

### Virus and stereotactic injection

Anesthesia was initiated with intraperitoneal injection of 1% pentobarbital sodium in 0.9% saline at 0.1 mL/20 g body weight. After the animals were deeply anesthetized, the eyes were covered with eye ointment to protect them from drying and injury. The animal was mounted on a stereotaxic frame with ear bars. A small incision (1 cm in length) was made in the skin above the surgical site with sterilized stainless-steel surgical scissors, and the skin and fascia were removed to expose the skull. Coordinates were measured with a stereotaxic instrument, and a small hole was drilled through the skull with a dental drill. For retrograde or anterograde experiments, tracers were delivered via glass micropipettes mounted on a Nanoject II (Drummond Scientific) using a pressure injection pump at a speed of 40 nL/min. After the injections, the skin was sutured, lidocaine hydrochloride gel was applied to the wound, and the mice were returned to their home cages for recovery.

For retrograde monosynaptic tracing, 150 nL of a 2:1 mixture of rAAV9-EF1α-DIO-RG-WPRE-pA and rAAV2/9-Ef1α-DIO-mCherry-2a-TVA-WPRE-pA (Titer: 2.00E + 12 vg/ml, virus from BrainVTA) virus was injected into the target mPFC regions. The following coordinates (mm from bregma) were used: anterior PL (aPL): AP: + 2.68, ML: + 0.4 (for Fezf2)/ + 0.25 (for Plxnd1), DV: − 1.7; middle PL (mPL): AP: + 2.22, ML: + 0.4/ + 0.25, DV: − 2.15; posterior PL (pPL): AP: 1.75, ML: + 0.4/ + 0.25, DV: − 2.3; ILA: AP: + 1.65, ML: + 0.4/ + 0.25, DV: − 2.7. Three days later, 20 mg/kg TM (Sigma, T5648-1G) was intraperitoneally administered to the mice, resulting in transgene recombination. Three weeks later, 250 nL of RV-△G-EnvA-eGFP (2 × 10^8^ vg/mL, from BrainVTA) was injected into the same region, and 7 days later, the mice were sacrificed through CO2 inhalation. For axonal AAV tracing, 100 nL of rAAV2/5-EF1α-DIO-eGFP-WPRE-pA (2 × 10^12^ vg/ml, virus from BrainVTA) was injected into a target mPFC region at the above coordinates. Three days later, 20 mg/kg TM was intraperitoneally injected into the mice. Three weeks later, the mice were sacrificed through CO2 inhalation.

### Histology

Animals were deeply anesthetized with pentobarbital sodium and perfused intracardially with 0.01 M phosphate buffered solution (PBS, Sigma-Aldrich), followed by 4% paraformaldehyde (PFA, Sigma-Aldrich) in 0.01 M PBS. The brains were postfixed for an additional 12 h in 4% PFA at 4 °C. To obtain whole-brain imaging, the brain was embedded in glycol methacrylate (GMA) resin (Ted Pella Inc.), and the embedding protocol has been previously described [60, 61]. Briefly, each intact brain was rinsed overnight at 4 °C in 0.1 mol/L PBS, and it was dehydrated in a graded ethanol series (50, 70, and 95% ethanol, changing from one concentration to the next every 1 h at 4 °C). Subsequently, the brains were immersed in a graded GMA resin, including 0.2% Sudan Black B (70, 85, and 100% GMA for 2 h each and 100% GMA overnight at 4 °C). Finally, the samples were immersed in GMA solution for 3 days at 4 °C and embedded in a vacuum oven at 48 °C for 24 h. During the experiment, the mice were euthanized by inhalation of CO_2_ when necessary.

### Imaging and preprocessing

To obtain whole-brain high resolution imaging, a dual-color fMOST imaging system was used (the output samples were stained with propidium iodide) on the GMA resin-embedded samples developed by our group [32]. In brief, the embedded sample was mounted on a high-precision 3D translation stage. By moving the stage, the lens imaged the entire plane of the sample in a mosaic manner. Then, a diamond knife was used to remove the imaged area, and a new image of the sample surface was taken. A complete and continuous mouse brain dataset could be obtained after many cycles of imaging with a voxel resolution of 0.32 μm × 0.32 μm × 2 μm. To generate full coronal sections, preprocessing, including mosaic stitching and illumination correction, was conducted on the acquired two-channel picture files.

### Data processing

To calculate the numbers of input neurons and the pixels of output fiber signals, we developed our approach [62, 63]. Briefly, the coordinates of the input neuron somata were obtained using NeuroGPS. The coordinates of the soma of the input neurons and a high-resolution picture stack of labeled outputs were registered to Allen CCFv3. Each registered output coronal slice was background removed, Gaussian filtered, and threshold segmented to binary images to identify the projection signal. To eliminate mistakes, all the results were manually examined. The number of somata or the volume of the projection signal was then quantified in each brain area. We calculated the proportion of connections in different regions to normalize the connection strength between the different samples.

### Visualization and statistical analysis

To visualize the input and output results, ImageJ, Amira (v6.1.1, FEI), Python 3.8.4, and MATLAB (v2017a, MathWorks) were used. All histograms and heatmaps were generated by GraphPad Prism (v.6.0, GraphPad) and SPSS (IBM SPSS Statistics 23). The histograms displayed all of the individual data points, and no data points were removed from the analysis.

We performed multiple unpaired two-sided Student’s *t* tests to generate *P* values to assess the differences in connection strength across various brain areas, with the confidence level set to 0.05 (*P* value), and all results are presented as the mean ± SEM. No correction was applied for multiple comparisons. Circles in the bar graphs represent individual animals.

Pearson correlation coefficients and hierarchical cluster analysis were used to examine the similarities and differences in the strength of connections between various samples or brain regions. In the drawing of the correlation heatmap, firstly, the Pearson correlation coefficients between the group data were calculated by Prism, and then all Pearson correlation coefficients were hierarchically clustered by SPSS. According to the clustering results, the original data in Prism were reordered, and the Pearson correlation coefficient matrix between the reordered data was calculated again. Based on that, the corresponding hierarchical clustering heatmap was drawn by Prism. Each row and each column refer to the Pearson correlation coefficient between the corresponding two samples or brain regions. Data from Fezf2 inputs: *n* = 5 (aPL), *n* = 7 (mPL and pPL), *n* = 3 (ILA); Plxnd1 inputs: *n* = 4 (aPL and mPL), *n* = 5 (pPL), *n* = 9 (ILA); Fezf2 outputs: *n* = 4 (aPL, mPL and ILA), *n* = 3 (pPL); Plxnd1 outputs: *n* = 5 (aPL), *n* = 3 (mPL, and ILA), *n* = 4 (pPL).

### Supplementary Information

**Additional file 1:** **Figure S1.** The distribution of starter cells. **Figure S2.** Schematic illustration showing the starter cell distribution center. **Figure S3.** The connection pattern of the whole brain input and output. **Figure S4.** Representative continuous coronal images of inputs to Fezf2 and Plxnd1 neurons in mPFC subregions. **Figure S5.** Representative continuous coronal images of the outputs of Fezf2 and Plxnd1 neurons in mPFC subregions. **Figure S6.** Schematic coronal sections depicting input neurons and output fibers of Fezf2 and Plxnd1 neurons in mPFC subregions. **Figure S7.** The proportion of the input neurons of Fezf2 and Plxnd1 neurons in the mPFC subregions in discrete brain regions. **Figure S8.** Comparisons of inputs to Plxnd1 and Fezf2 neurons in the mPFC subregions. **Figure S9.** Brain-wide output datasets of Fezf2 neurons in the mPFC subregions. **Figure S10.** Brain-wide output datasets of Plxnd1 neurons in the mPFC subregions. **Figure S11.** Brain regions with significant differences in the proportions of input and output. **Figure S12.** Laminar distribution of cortical input neurons to Fezf2 neurons in mPFC subregions. **Figure S13.** Laminar distribution of cortical input neurons of Plxnd1 neurons in the mPFC. **Figure S14.** The mPFC-thalamic connectivity. **Figure S15.** Comparisons of thalamic inputs to and outputs of Fezf2 neurons in the mPFC subregions. **Figure S16.** mPFC-amygdala connectivity. **Figure S17.** mPFC-BLAa connectivity. **Figure S18.** mPFC-hypothalamic connectivity. **Figure S19.** The axon terminals of the reconstructed single neurons. **Figure S20.** Brain regions with significant differences in the proportion of input between PL subregions. **Figure S21.** The correlation between the mPFC subregions according to their input-output connections. **Table S1.** More detailed abbreviations list.

**Additional file 2.** Fezf2 and Plxnd1 input and output raw data.

## Data Availability

All data generated or analyzed during this study are included in this published article and its supplementary information files.

## Abbreviations

3D: Three-dimensional
AId: Agranular insular area, dorsal part
AIp: Agranular insular area, posterior part
AIV: Agranular insular cortex, ventral part
AM: Anteromedial nucleus of the thalamus
A-P: Anterior-posterior
aPL: Prelimbic area, anterior part
ARA: Allen Reference Atlas
ATN: Anterior thalamic nuclei
BLA: Basolateral amygdalar nucleus
BLAa: Basolateral amygdalar nucleus, anterior part
BMA: Basomedial amygdalar nucleus
CA1d: Dorsal of CA1
CA1i: Intermediate of CA1
CA1v: Ventral of CA1
CL: Central lateral nucleus of the thalamus
CM: Central medial nucleus of the thalamus
COA: Cortical amygdala area
CP: Caudoputamen
CT: Corticothalamic
DP: Dorsal peduncular area
ECT: Ectorhinal area
ENTl: Entorhinal area, lateral part
FF: Fields of Forel
fMOST: Fluorescence micro-optical sectioning tomography
FRP: Frontal pole
GMA: Glycol methacrylate
GPe: Globus pallidus, external segment
GPi: Globus pallidus, internal segment
GU: Gustatory areas
HPF: Hippocampal formation
IAD: Interanterodorsal nucleus of the thalamus
ILA: Infralimbic area
ILM: Intralaminar thalamic nuclei
IMD: Intermediodorsal nucleus of the thalamus
IT: Intratelencephalic
LD: Lateral dorsal nucleus of the thalamus
LHA: Lateral hypothalamic area
LP: Lateral posterior nucleus of the thalamus
LPO: Lateral preoptic area
MA: Magnocellular nucleus
MD: Mediodorsal nucleus of the thalamus
MEA: Medial amygdalar nucleus
MED: Medial dorsal thalamic nuclei
MOp: Primary motor area
MOs: Secondary motor area
mPFC: Medial prefrontal cortex
mPL: Prelimbic area, middle part
MS: Medial septal nucleus
MTN: Midline thalamic nuclei
NDB: Diagonal band nucleus
ORB: Orbital area
ORBm: Orbital area, medial part
PAG: Periaqueductal gray
PCN: Paracentral nucleus of the thalamus
PERI: Perirhinal area
PH: Posterior hypothalamic nucleus
PIR: Piriform area
PL: Prelimbic area
pPL: Prelimbic area, posterior part
PSTN: Parasubthalamic nucleus
PT: Parataenial nucleus
PVT: Paraventricular thalamic nucleus
RE: Nucleus of reunions
RSP: Retrosplenial area
RSPagl: Retrosplenial area, lateral agranular part
RSPv: Retrosplenial area, ventral part
RT: Reticular nucleus of the thalamus
SI: Substantia innominata
SMT: Submedial nucleus of the thalamus
STN: Subthalamic nucleus
SUB: Subiculum
TM: Tamoxifen
TT: Taenia tecta
VAL: Ventral anterior-lateral complex of the thalamus
VENT: Ventral thalamic nuclei
VIS: Visual areas
VM: Ventral medial nucleus of the thalamus
ZI: Zona incerta
