# Supplementary material for: Subregion preference in the long-range connectome of pyramidal neurons in the medial prefrontal cortex
Source: BMC Biol. 2024 Apr 29;22:95. doi: 10.1186/s12915-024-01880-7 (PMC11057135; doi:10.1186/s12915-024-01880-7)
Supplement: Supplementary file 1 — Additional file 1: Figure S1. The distribution of starter cells. Figure S2. Schematic illustration showing the starter cell distribution center. Figure S3. The connection pattern of the whole brain input and output. Figure S4. Representative continuous coronal images of inputs to Fezf2 and Plxnd1 neurons in mPFC subregions. Figure S5. Representative continuous coronal images of the outputs of Fezf2 and Plxnd1 neurons in mPFC subregions. Figure S6. Schematic coronal sections depicting input neurons and output fibers of Fezf2 and Plxnd1 neurons in mPFC subregions. Figure S7. The proportion of the input neurons of Fezf2 and Plxnd1 neurons in the mPFC subregions in discrete brain regions. Figure S8. Comparisons of inputs to Plxnd1 and Fezf2 neurons in the mPFC subregions. Figure S9. Brain-wide output datasets of Fezf2 neurons in the mPFC subregions. Figure S10. Brain-wide output datasets of Plxnd1 neurons in the mPFC subregions. Figure S11. Brain regions with significant differences in the proportions of input and output. Figure S12. Laminar distribution of cortical input neurons to Fezf2 neurons in mPFC subregions. Figure S13. Laminar distribution of cortical input neurons of Plxnd1 neurons in the mPFC. Figure S14. The mPFC-thalamic connectivity. Figure S15. Comparisons of thalamic inputs to and outputs of Fezf2 neurons in the mPFC subregions. Figure S16. mPFC-amygdala connectivity. Figure S17. mPFC-BLAa connectivity. Figure S18. mPFC-hypothalamic connectivity. Figure S19. The axon terminals of the reconstructed single neurons. Figure S20. Brain regions with significant differences in the proportion of input between PL subregions. Figure S21. The correlation between the mPFC subregions according to their input-output connections. Table S1. More detailed abbreviations list. [file 12915_2024_1880_MOESM1_ESM.docx]

**Additional File 1. Figures S1-S21**


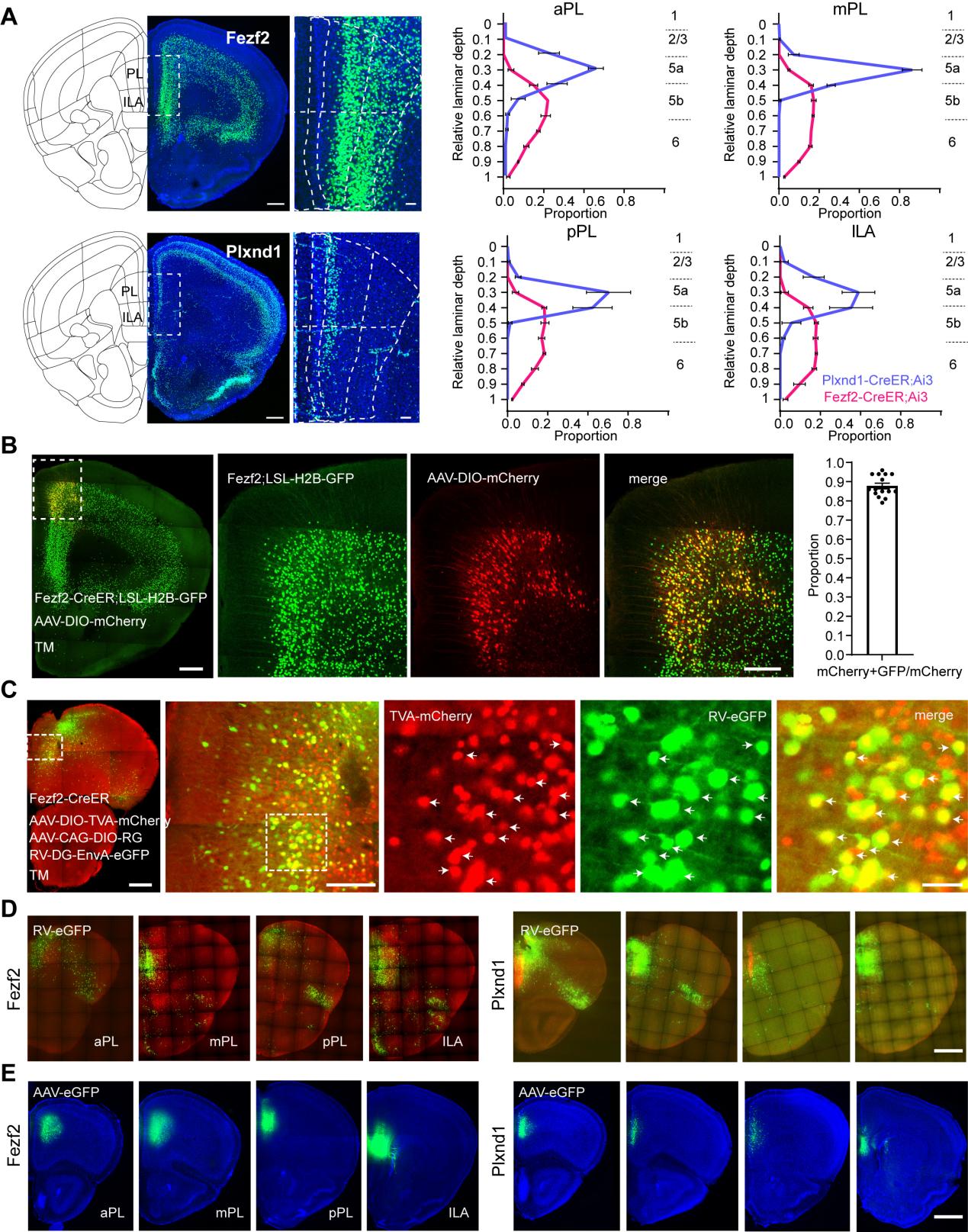


Figure S1: The distribution of starter cells. (A) Left panel, distribution of Fezf2- and Plxnd1- positive neurons in Fezf2-CreER;LSL-H2B-GFP and Plxnd1-CreER; LSL-H2B-GFP transgenic hybrid mice. Cytoarchitecture information was obtained by 4,6-diamino-2-phenyl indole (DAPI) staining. The white boxes correspond to the PL and ILA regions. The columns on the right are the high-magnification images of the white frame on the coronal plane. Scale bars: left, 500 µm. right, 100 µm. Right panel, average soma distribution in the mPFC of two transgenic mice (Fezf2-CreER;Ai3 and Plxnd1-CreERL;Ai3). The relative laminar depth refers to the distance from the surface of the cortex to the white matter. The data are displayed as the average ±SEM. Data from Fezf2 and Plxnd1: n = 3 per group. (B) Validation of the specificity of virus labeling. AAV-DIO-mCherry was performed in the mPFC of Fezf2-CreER;LSL-H2B-GFP mice. Scale bars: 500 µm and 200 µm. (C) Display of starter cells in the mPFC. Right: magnification of the neurons on the left. Arrows indicate starter cells co-labeled with RV-eGFP and TVA-mCherry. Scale bars: 500 µm, 200 µm, and 50 µm. (D) Demonstration of the four mPFC injection sites of input tracing. Scale bars: 1 mm. (E) Demonstration of the injection site of output tracing. Scale bars: 1 mm.


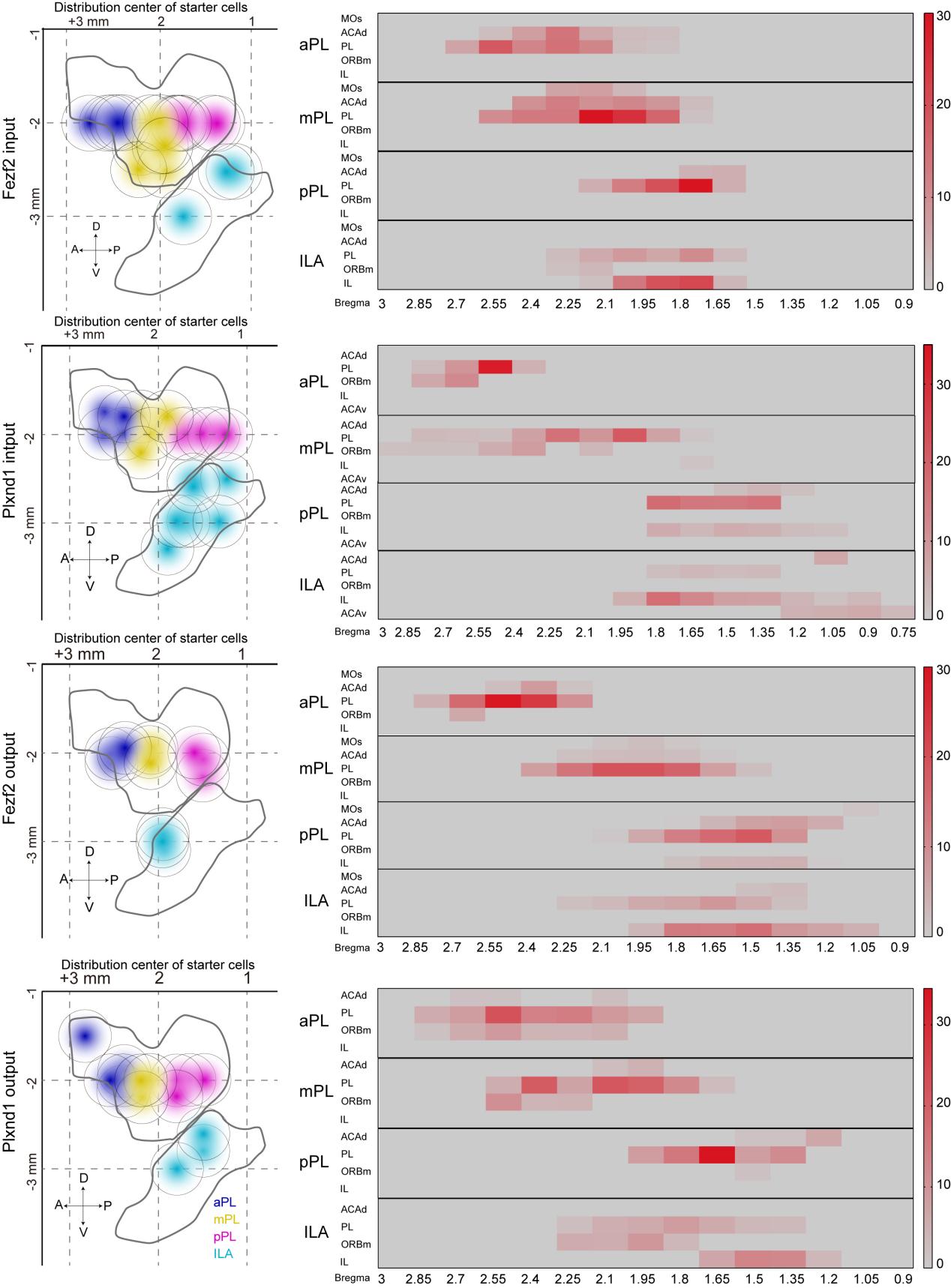


Figure S2: Schematic illustration showing the starter cell distribution center. Schematic illustration of the lateral view of the mPFC showing the starter cell distribution center (left panel), and heatmaps showing the average starter cell distribution along the A-P axis for each tracing strategy at each specific mPFC site (right panel).


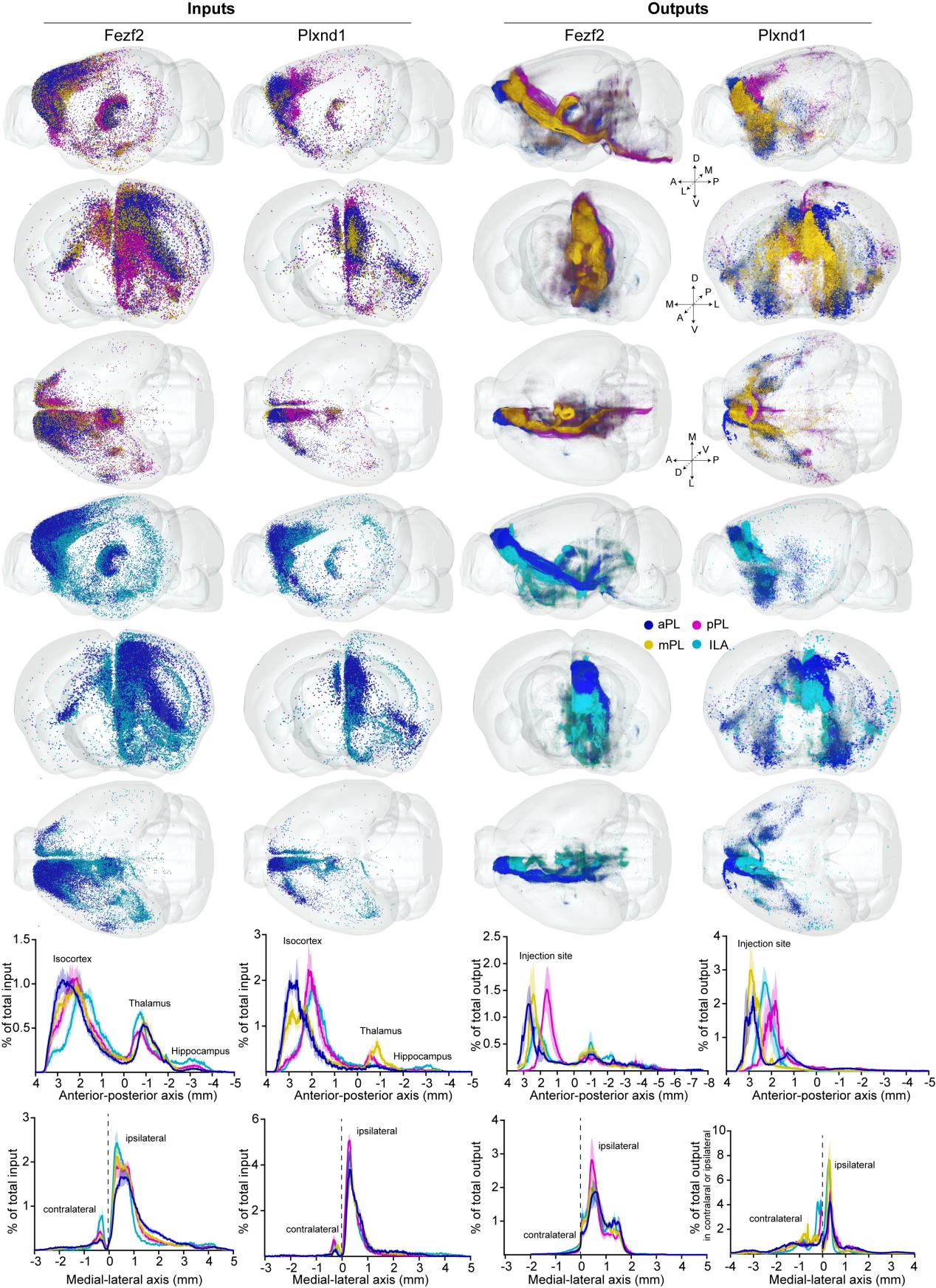


Figure S3: The connection pattern of the whole brain input and output. The upper column shows a 3D schematic depicting the detected input neurons and output fibers of Plxnd1 and Fezf2 neurons. In the lower column, the input cell density and output fiber density plot along the A-P axis and dorsal-ventral axis. The density plot data are displayed as the average ± SEM, and the SEM is indicated by the shaded area. Data are from Fezf2 inputs: n = 5 (aPL), n = 7 (mPL and pPL), n = 3 (ILA); Plxnd1 inputs: n = 4 (aPL and mPL), n = 5 (pPL), n = 9 (ILA); Fezf2 outputs: n = 4 (aPL, mPL, and ILA), n = 3 (pPL); Plxnd1 outputs: n = 5 (aPL), n = 3 (mPL and ILA), n = 4 animals (pPL).


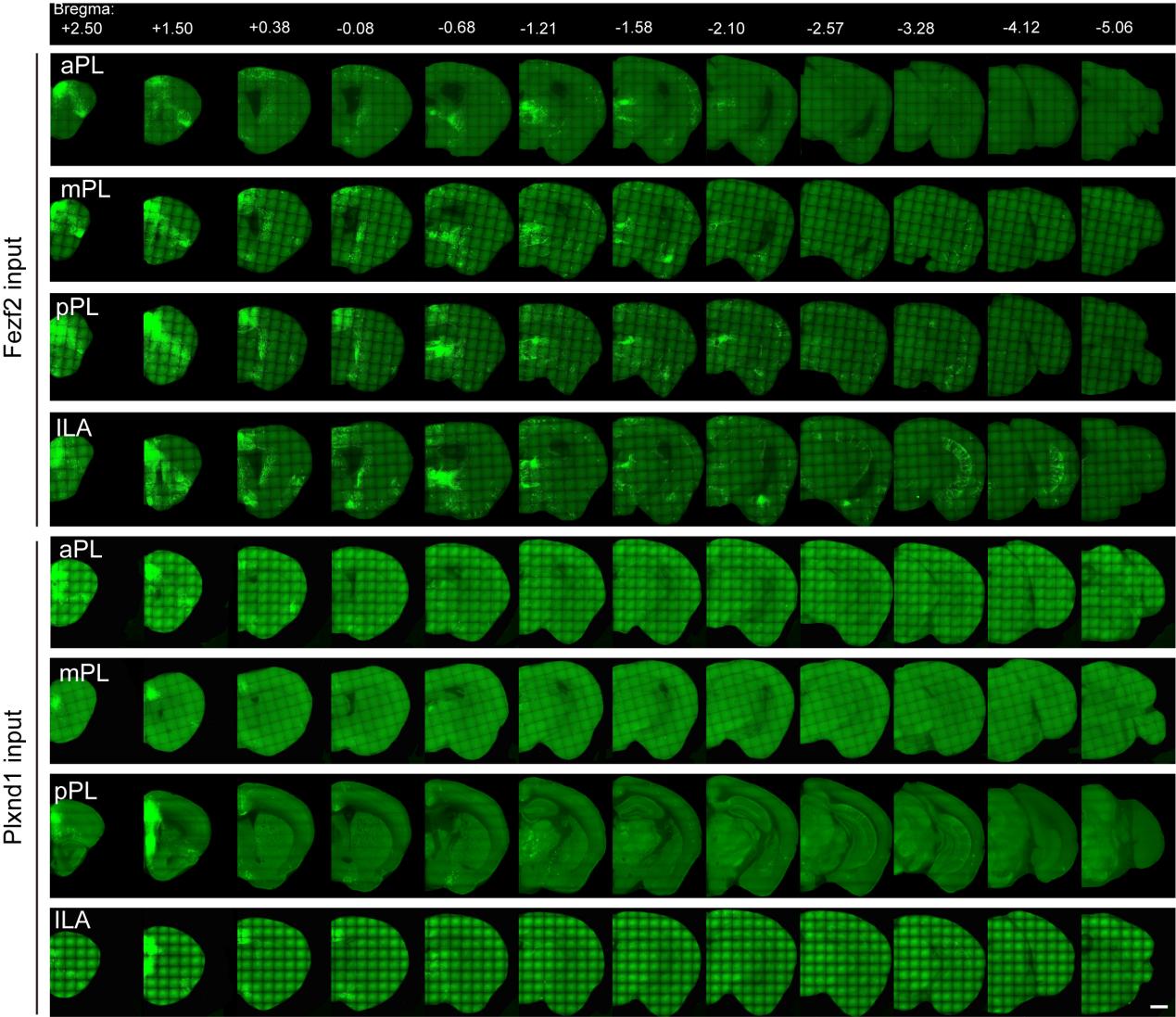


Figure S4: Representative continuous coronal images of inputs to Fezf2 and Plxnd1 neurons in mPFC subregions. Slice thickness, 50 μm. Scale bars, 1 mm.


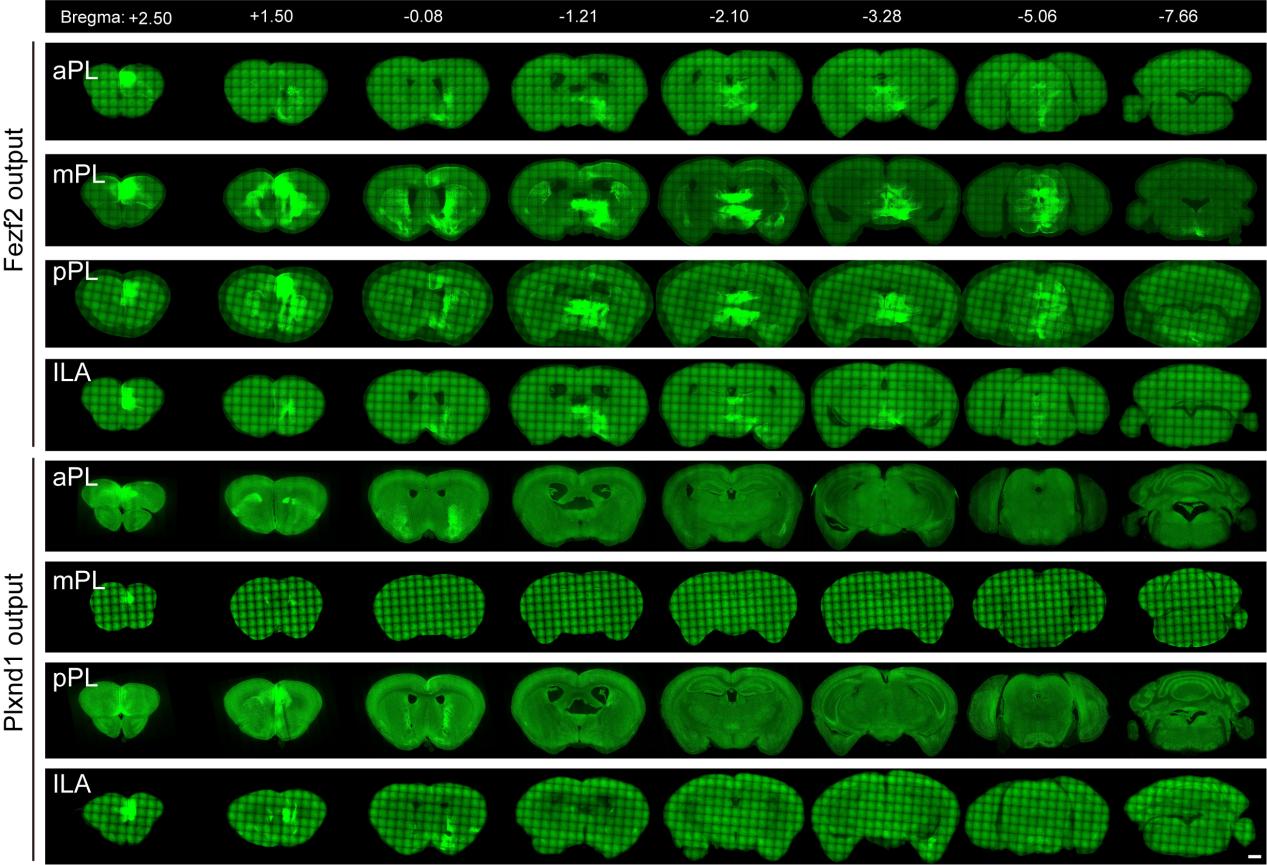


Figure S5: Representative continuous coronal images of the outputs of Fezf2 and Plxnd1 neurons in mPFC subregions. Slice thickness, 50 μm. Scale bars, 1 mm.


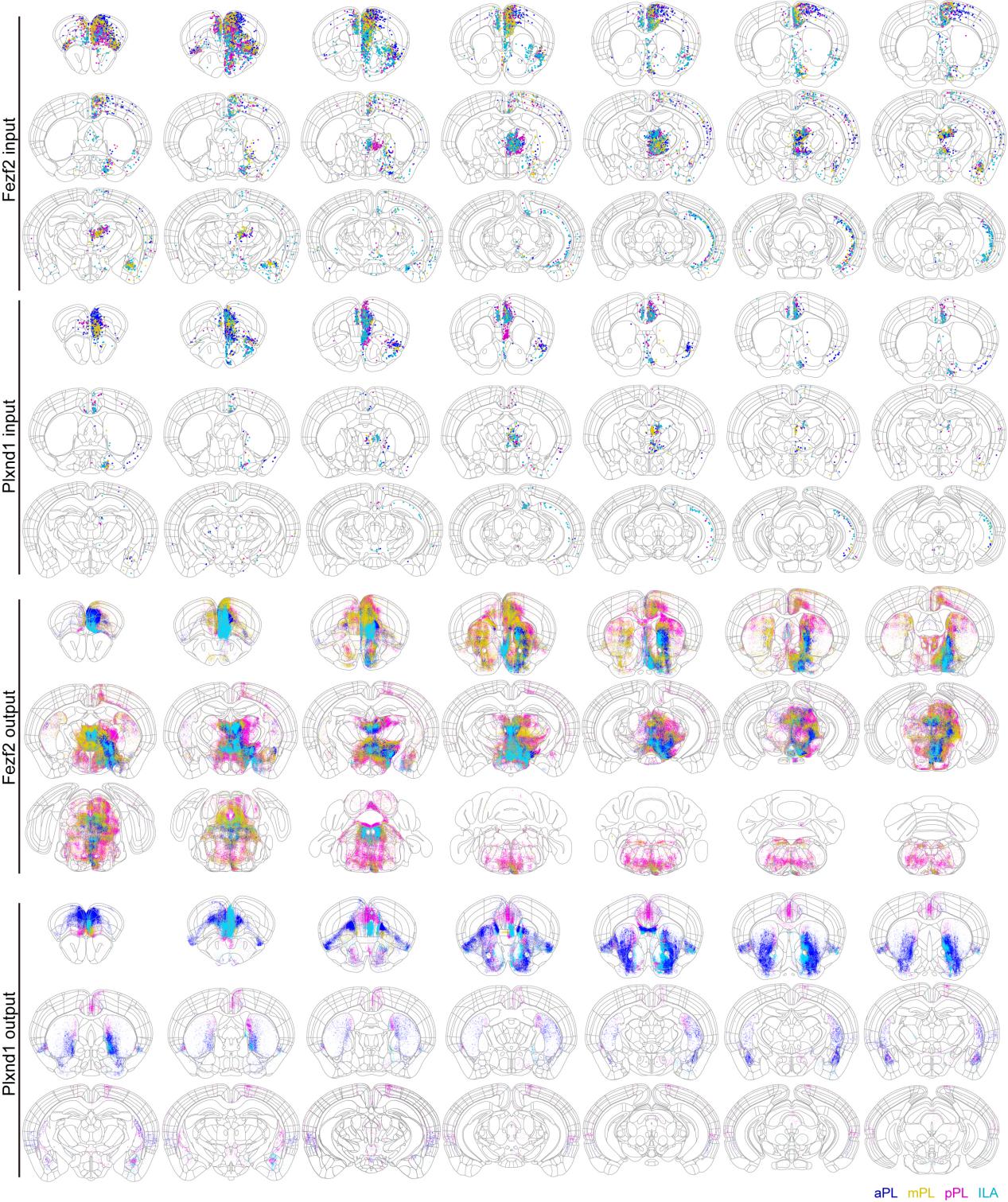


Figure S6: Schematic coronal sections depicting input neurons and output fibers of Fezf2 and Plxnd1 neurons in mPFC subregions (aPL, blue; mPL, yellow; pPL, purple; ILA, cyan). One dot represents one neuron. Slice thickness, 100 μm.


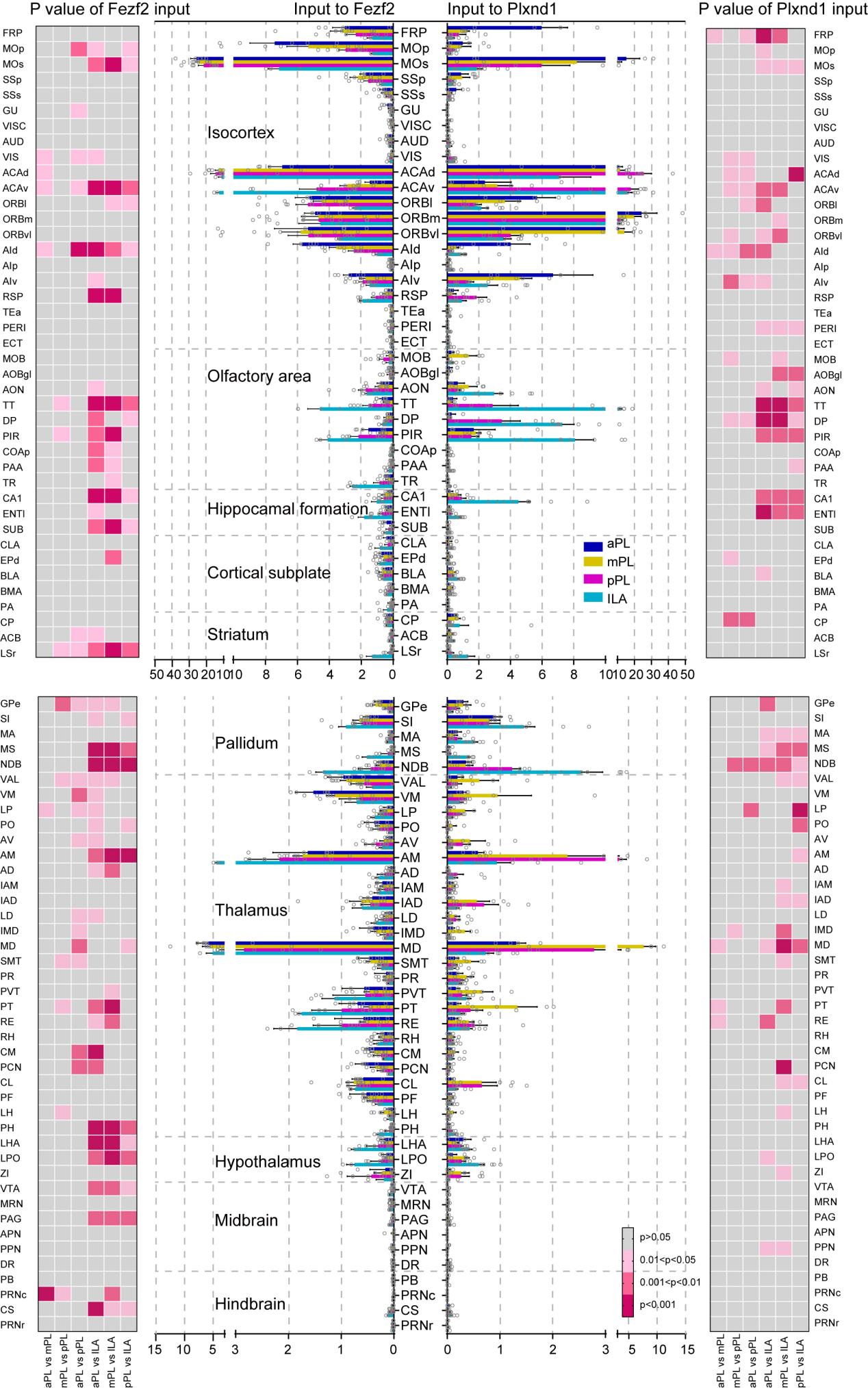


Figure S7: The proportion of the input neurons of Fezf2 and Plxnd1 neurons in the mPFC subregions in discrete brain regions. The input neurons were detected mainly in 83 brain regions according to the brain structure in the ARA. The values are presented as normalized percentages of the total number of input cells. P value heatmap between the mPFC subregions. Two-sided Student’s t tests were used to generate P values. The data are shown as the mean ± SEM. Data from Fezf2 inputs: n = 5 (aPL), n = 7 (mPL and pPL), n = 3 (ILA); Plxnd1 inputs: n = 4 (aPL and mPL), n = 5 (pPL), n = 9 animals (ILA). For detailed abbreviations, see Additional file 1: Table S1.


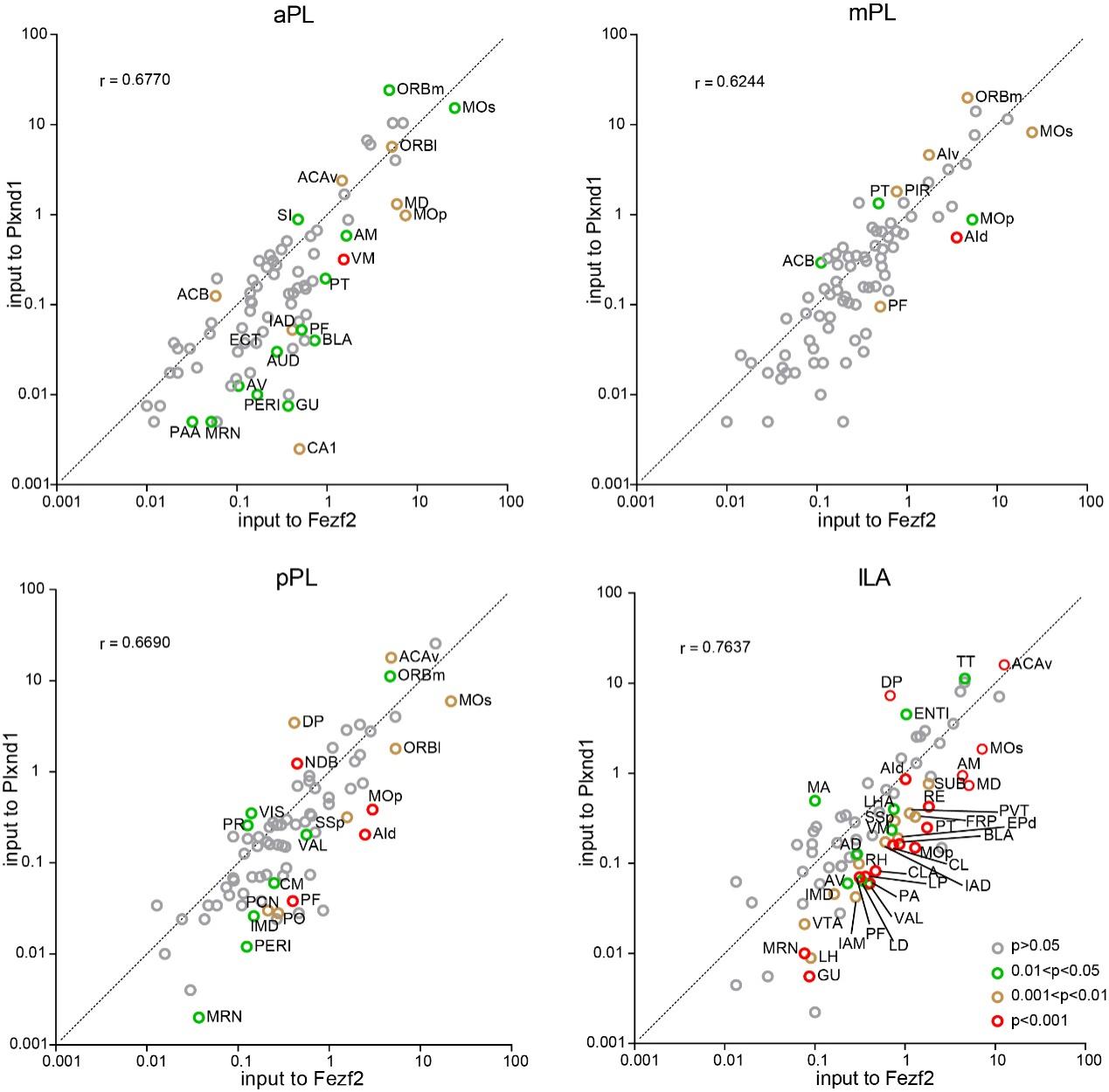


Figure S8: Comparisons of inputs to Plxnd1 and Fezf2 neurons in the mPFC subregions. The circles represent the proportion of input neurons in each brain region, where the color of the circles indicates significant differences (the colors of the circles: gray, green, yellow, and red indicated P >0.05, 0.01 < P < 0.05, 0.001 < P < 0.01, and P < 0.001, respectively). Two-sided Student’s t tests were used to generate P values. r: Pearson correlation coefficient. The P values were calculated using the proportions of the 83 input brain regions shown in Additional file 1: Fig. 1-S7. For detailed abbreviations, see Additional file 1: Table S1.


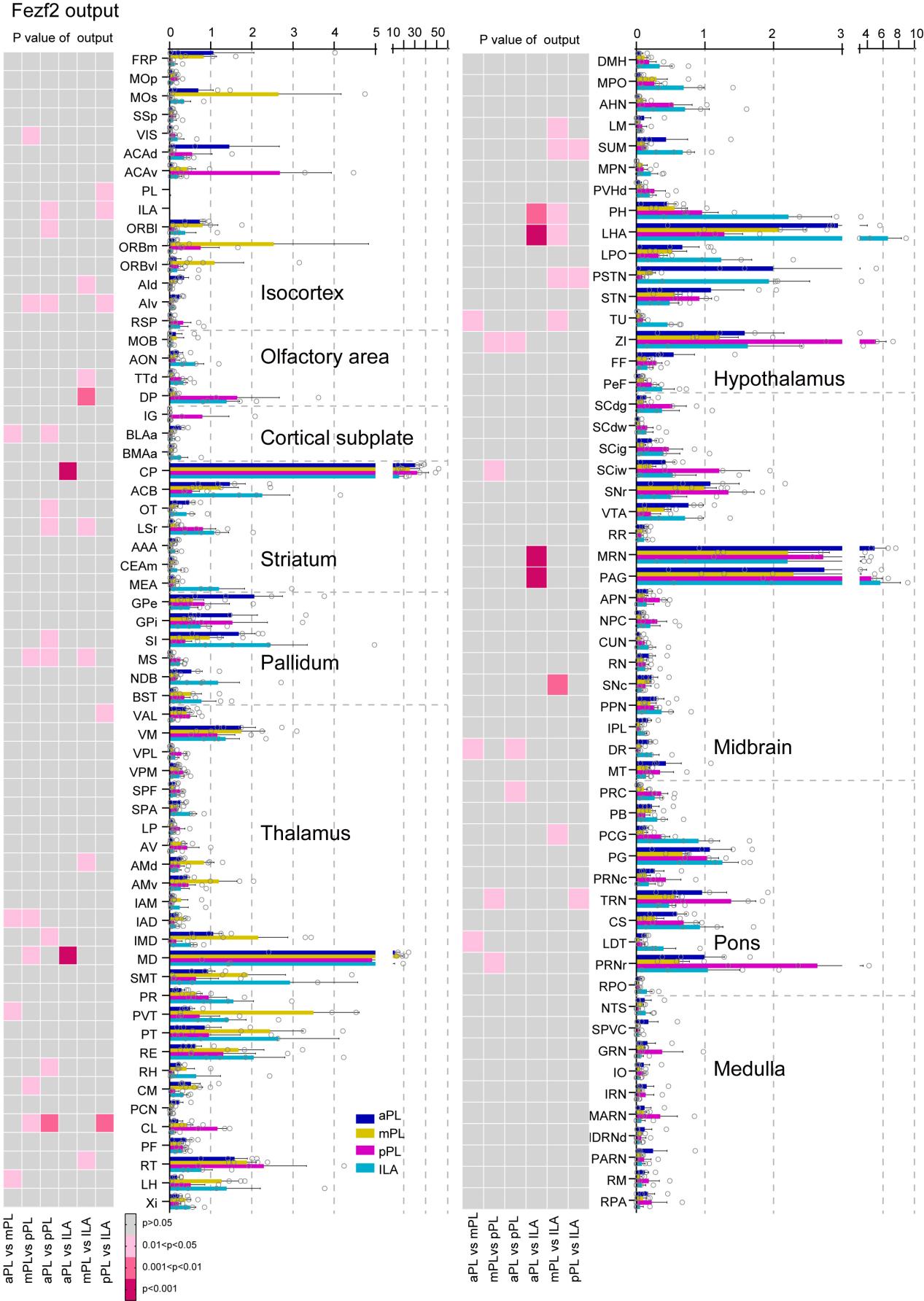


Figure S9: Brain-wide output datasets of Fezf2 neurons in the mPFC subregions. The output fibers were detected mainly in 116 brain regions according to the brain structure in the ARA. The values are presented as the normalized percentage of total pixels. P value heatmap between the mPFC subregions. Two-sided Student’s t tests were used to generate P values.. The data are shown as the mean ± SEM. Data from Fezf2 outputs: n = 4 (aPL, mPL and ILA), n = 3 animals (pPL). For detailed abbreviations, see Additional file 1: Table S1.


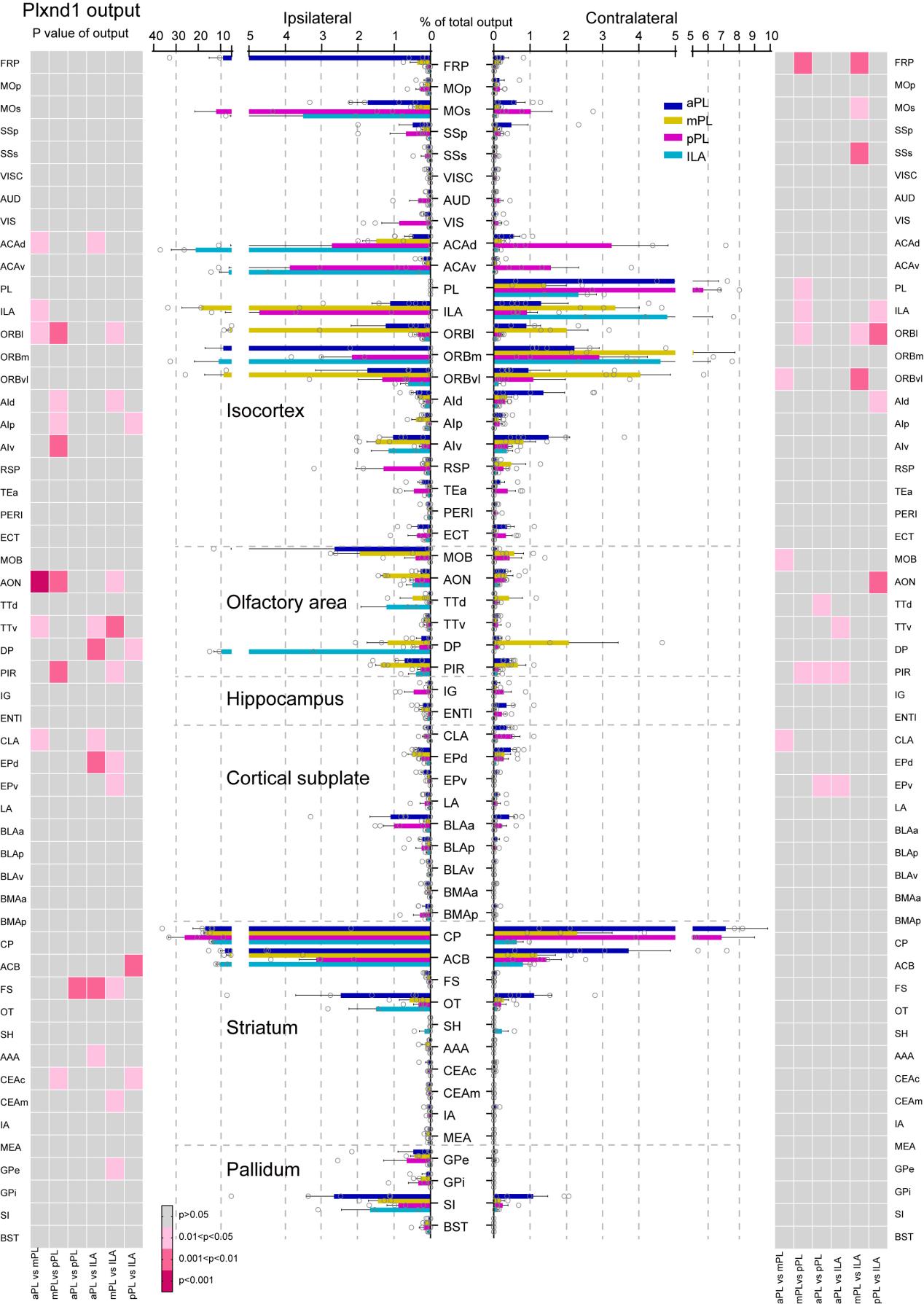


Figure S10: Brain-wide output datasets of Plxnd1 neurons in the mPFC subregions. The output fibers were detected mainly in 53 discrete brain regions according to the brain structure in the ARA. The values are presented as the normalized percentage of total output pixels. P value heatmap between the mPFC subregions. Two-sided Student’s t tests were used to generate P values. Data shown as the mean ± SEM. Data from Plxnd1 outputs: n = 5 (aPL), n = 3 (mPL, and ILA), n = 4 animals (pPL). For detailed abbreviations, see Additional file 1: Table S1.


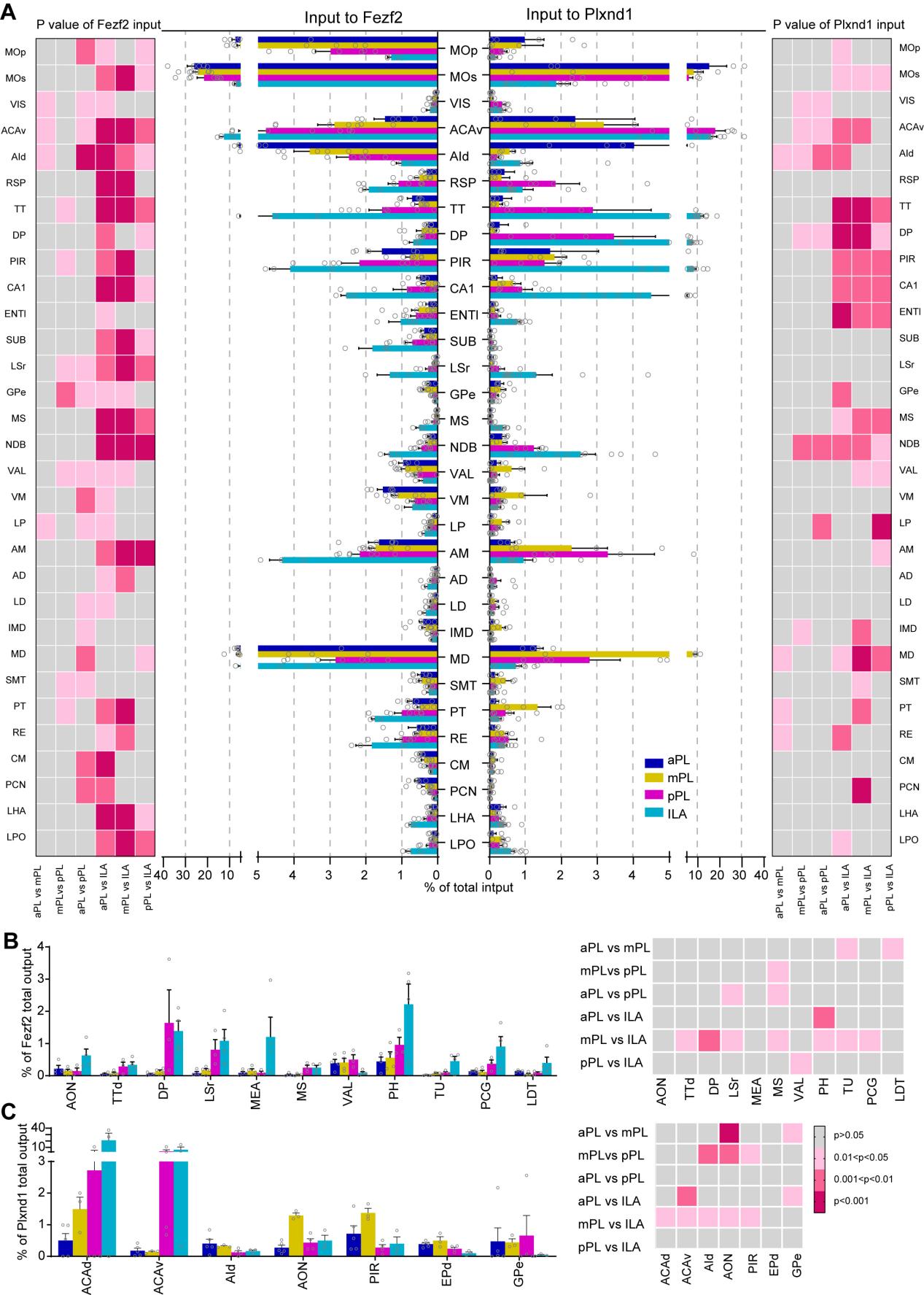


Figure S11: Brain regions with significant differences in the proportions of input and output. (A) The left panel shows brain regions that differentially innervated Plxnd1 or Fezf2 neurons in the mPFC subregions. Right panel, p value heatmap between the mPFC subregions. (B,C) Left panel, brain regions that Plxnd1 or Fezf2 neurons of the mPFC subregions differentially projected to. Right panel, p value heatmap between the mPFC subregions. A two-sided Student’s t-test was used to generate P values. The data in (A-C) are shown as the mean ± SEM, and the data are from Fezf2 inputs: n = 5 (aPL), n = 7 (mPL and pPL), n = 3 (ILA); Plxnd1 inputs: n = 4 (aPL and mPL), n = 5 (pPL), n = 9 (ILA); Fezf2 outputs: n =4 (aPL, mPL and ILA), n = 3 (pPL); Plxnd1 outputs: n = 5 (aPL), n = 3 (mPL, and ILA), n = 4 animals (pPL). For detailed abbreviations, see Additional file 1: Table S1.


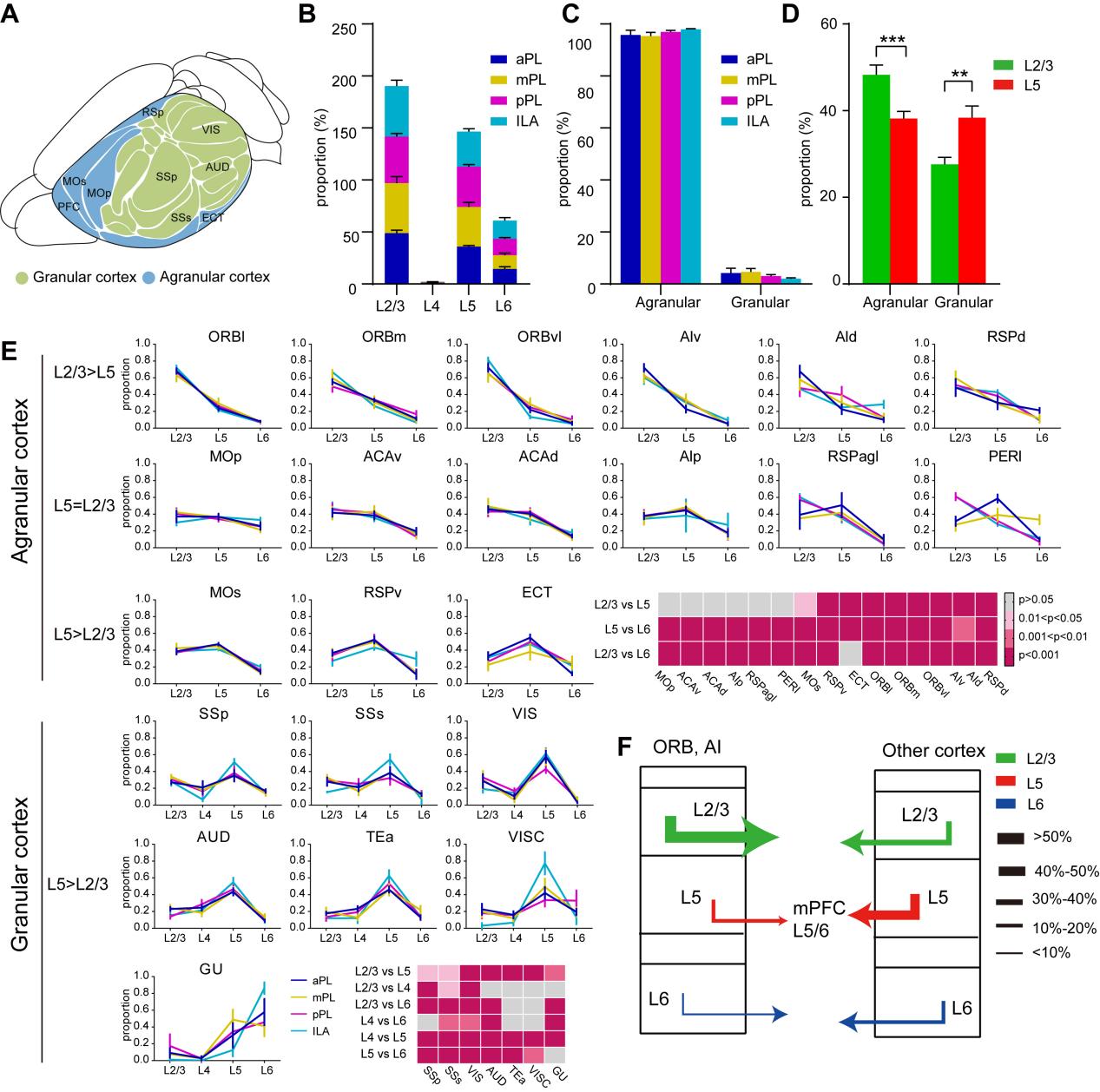


Figure S12: Laminar distribution of the cortical input neurons to Fezf2 neurons in mPFC subregions. (A) A three-dimensional view of the mouse isocortex showing the anatomical locations of the granular cortex and agranular cortex. (B) The cortical input neurons were mainly distributed in layers 2/3, followed by layer 5. (C) Cortical inputs of different mPFC subregions were mainly distributed in the agranular cortex. (D) Laminar distribution of cortical inputs in the granular cortex and agranular cortex. **P < 0.01, ***P < 0.001. The input neurons in the agranular cortex were mainly distributed in layers 2/3. The input neurons in the granular cortex were mainly distributed in layer 5. (E) Quantification of cortical input neurons to the distinct mPFC subregions in each cortical area. P value heatmap between the mPFC subregions. A two-sided Student’s t-test was used to generate P values. The data in (B-E) are displayed as the average ±SEM, and the data are from Fezf2 inputs: n = 5 (aPL), n = 7 (mPL and pPL), n = 3 (ILA) animals. For detailed abbreviations, see Additional file 1: Table S1.

_
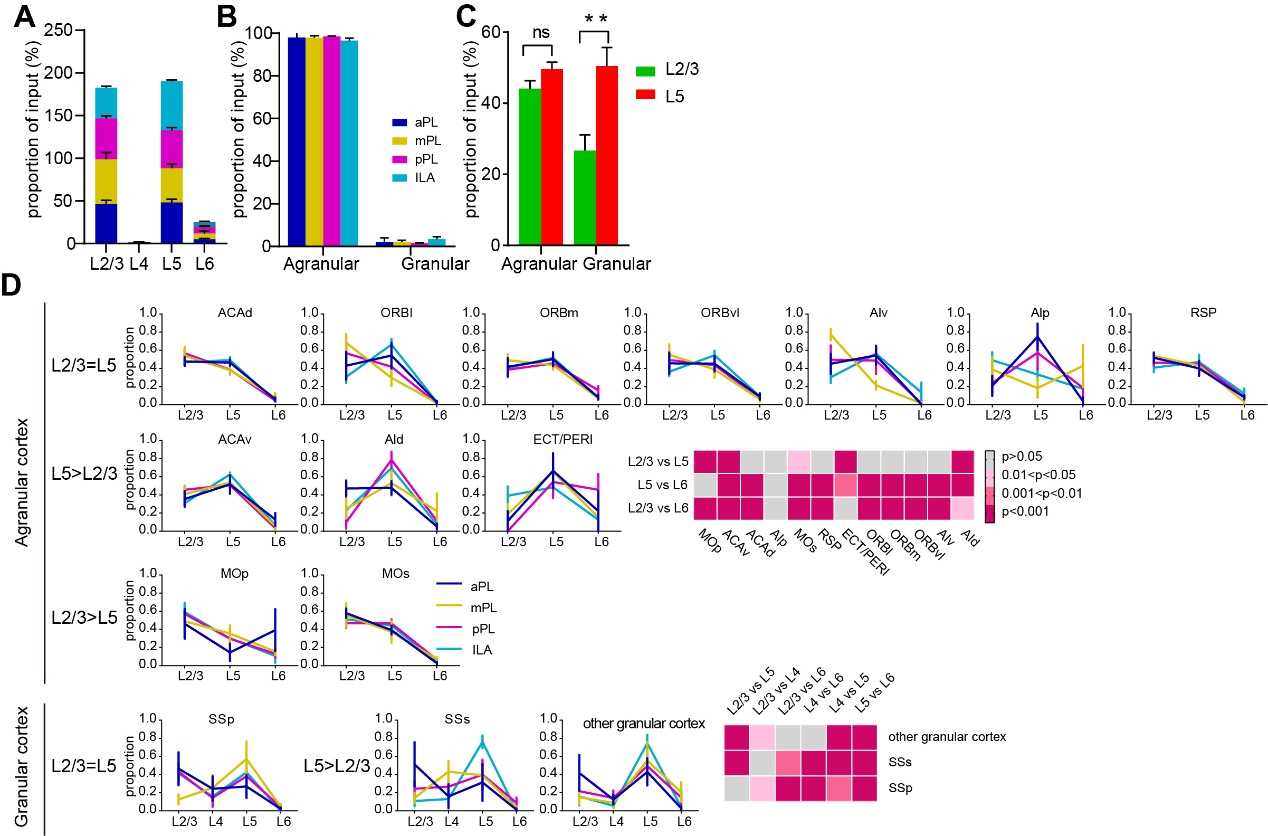
_

Figure S13: Laminar distribution of cortical input neurons of Plxnd1 neurons in the mPFC. (A) Cortical input neurons were mainly distributed in layers 2/3 and layer 5. (B) The cortical inputs of different mPFC subregions were distributed in the agranular cortex. (C) The laminar distribution of cortical inputs in the granular and agranular cortex. There was no difference between layer 2/3 and layer 5 of the input neurons in the agranular cortex. The input neurons in the granular cortex were mainly distributed in layer 5. 0.001 < ** P < 0.01. (D) Quantification of cortical input neurons to the distinct mPFC subregions in each cortical area. P value heatmap between the mPFC subregions. A two-sided Student’s t-test was used to generate P values. The data in (A-D) are displayed as the average ±SEM, and the data are from Plxnd1 inputs: n = 4 (aPL and mPL), n = 5 (pPL), n = 9 animals (ILA). For detailed abbreviations, see Additional file 1: Table S1.


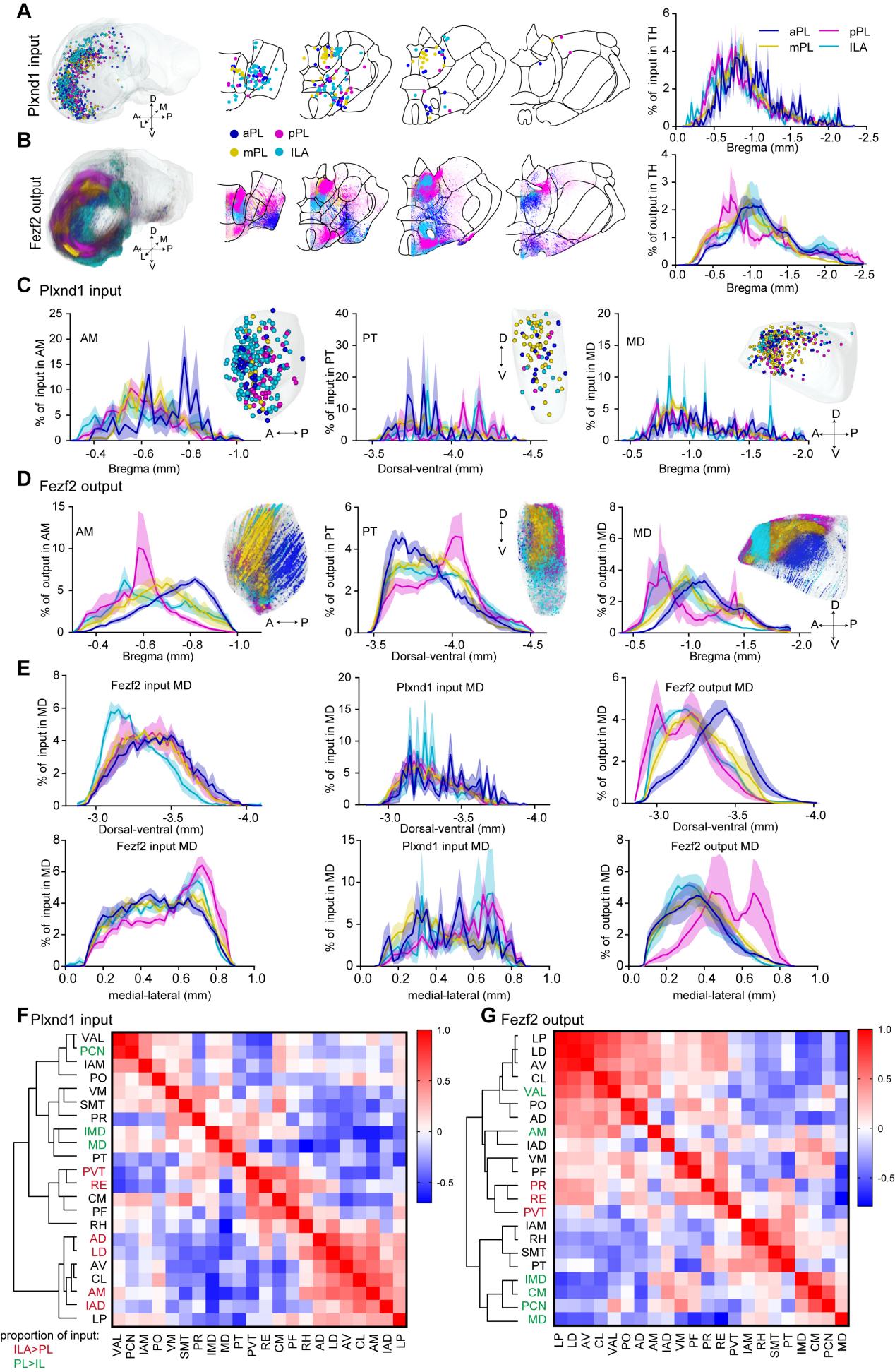


Figure S14: mPFC-thalamic connectivity. (A,B) Three-dimensional illustration and coronal sections (thickness: 100 μm) depicting detected input neurons to Plxnd1 neurons (A) or the output fibers from Fezf2 neurons (B) in the thalamus from representative samples. Different colors reflect inputs to or outputs from different mPFC subregions. Right panel, input cell density plot or output density plot along the A-P axis in the thalamus. (C,D) Comparison of the inputs in the AM, PT, and MD to Plxnd1 neurons (C) or outputs from Fezf2 neurons (D) in the different mPFC subregions. Right panel, three-dimensional images of input neurons or output fibers in the mPFC from representative samples. Left panel, density plot of input neurons or output fibers in the AM, PT, and MD along the A-P axis or dorsal-ventral axis. (E) Density plot of input neurons or output fibers in the MD along the dorsal-ventral axis or medial-lateral axis. (F,G) Pearson’s correlation coefficient matrix and hierarchal clustering investigating the clustering of thalamic nuclei according to their proportion of input to the mPFC subregions in Plxnd1 neurons and their proportion of output from Fezf2 neurons. The Pearson’s correlation coefficients were calculated using the input proportions in thalamic areas, as shown in Fig. 3A. The density plot data in (A-E) are displayed as the average ± SEM, and the SEM is indicated by the shaded area. Data from Fezf2 inputs: n = 5 (aPL), n = 7 (mPL and pPL), n = 3 (ILA); Plxnd1 inputs: n = 4 (aPL and mPL), n = 5 (pPL), n = 9 (ILA); Fezf2 outputs: n =4 (aPL, mPL and ILA), n = 3 animals (pPL). SEM is indicated by the shaded area. For detailed abbreviations, see Additional file 1: Table S1.


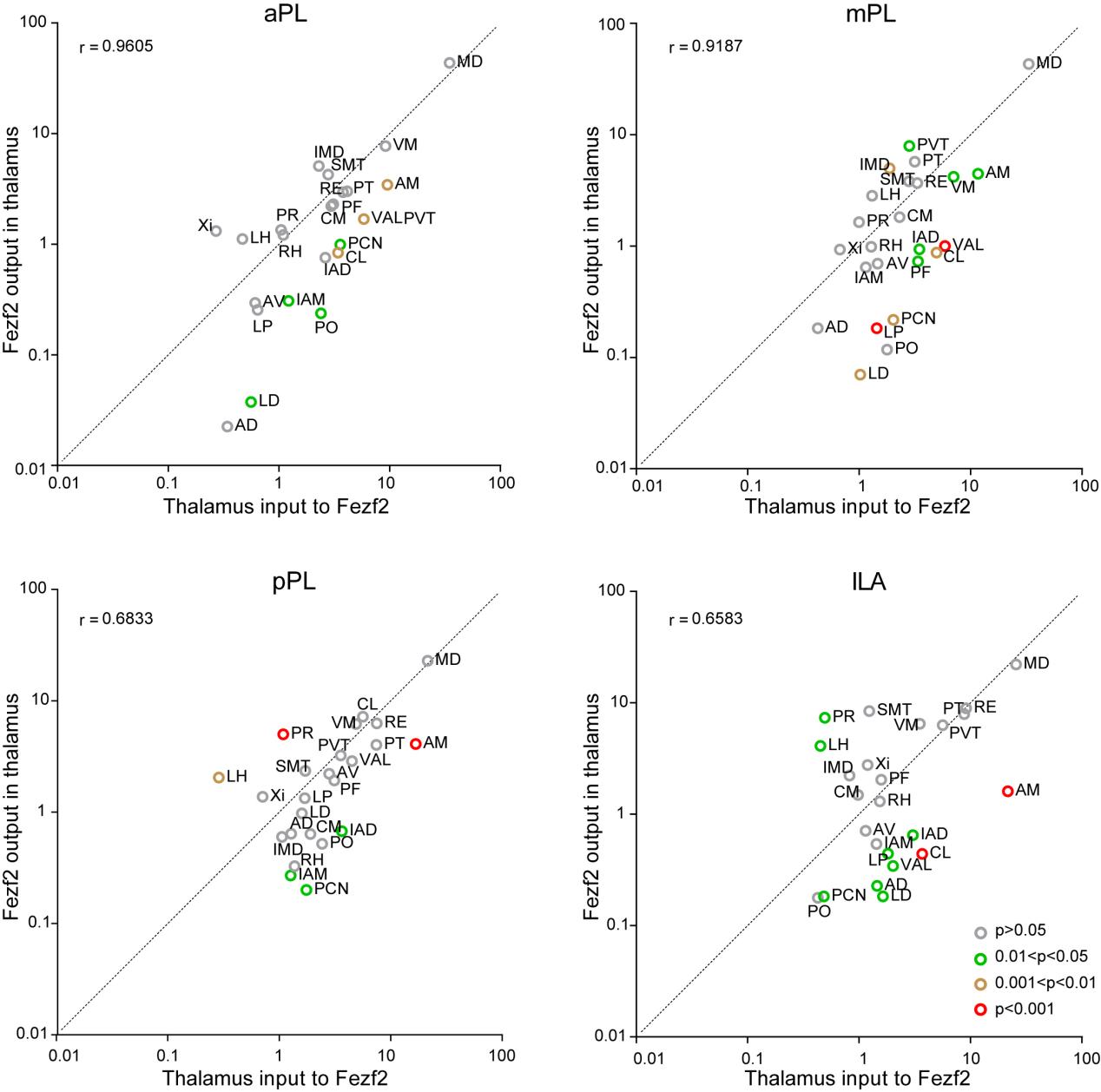


Figure S15: Comparisons of thalamic inputs to and outputs of Fezf2 neurons in the mPFC subregions. The circles represent the proportion of input neurons in each brain region, where the color of the circles indicates significant differences (the colors of the circles: gray, green, yellow, and red indicate P >0.05, 0.01 < P < 0.05, 0.001 < P < 0.01, and P < 0.001, respectively). Two-sided Student’s t tests were used to generate P values. r: Pearson correlation coefficient. The P values were calculated using the input and output proportions in the thalamic areas shown in Fig. 3A. For detailed abbreviations, see Additional file 1: Table S1.


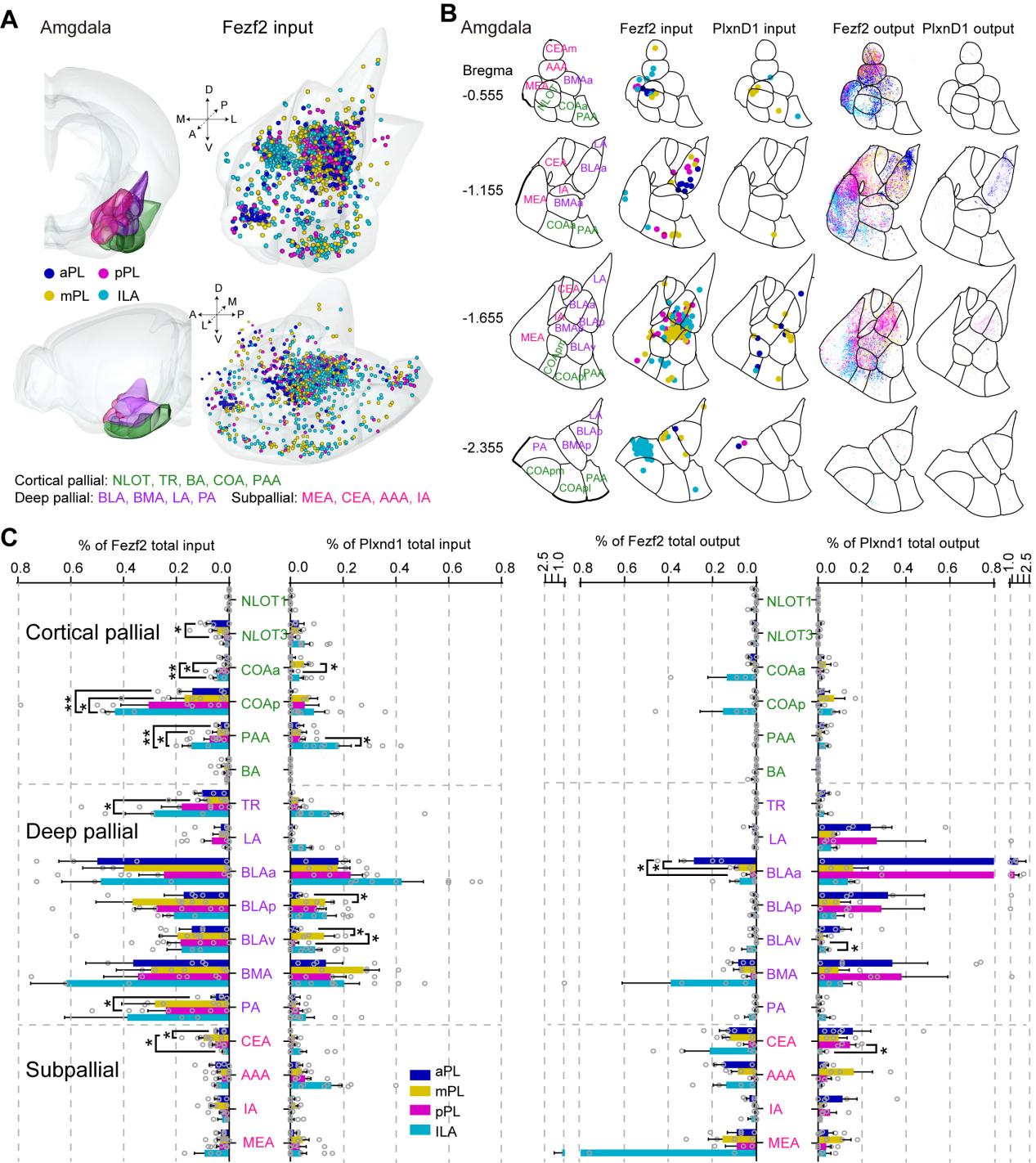


Figure S16: mPFC-amygdala connectivity. (A) Schematic illustration of the amygdala with its brain regions and three-dimensional illustration of amygdala inputs to the Fezf2 and Plxnd1 neurons in the mPFC subregions. (B) Schematic coronal sections (thickness: 100 μm) depicting amygdala inputs to and outputs from the Fezf2 and Plxnd1 neurons. (C) Proportions of input in discrete amygdalar regions. The data are displayed as the average ±SEM, and data from Fezf2 inputs: n = 5 (aPL), n = 7 (mPL and pPL), n = 3 (ILA); Plxnd1 inputs: n = 4 (aPL and mPL), n = 5 (pPL), n = 9 (ILA); Fezf2 outputs: n =4 (aPL, mPL and ILA), n = 3 (pPL); Plxnd1 outputs: n = 5 (aPL), n = 3 (mPL, and ILA), n = 4 animals (pPL). A two-sided Student’s t-test was used to generate P values. Significant differences were labeled as ***p<0.001, **p<0.01, and *p<0.05. For detailed abbreviations, see Additional file 1: Table S1.


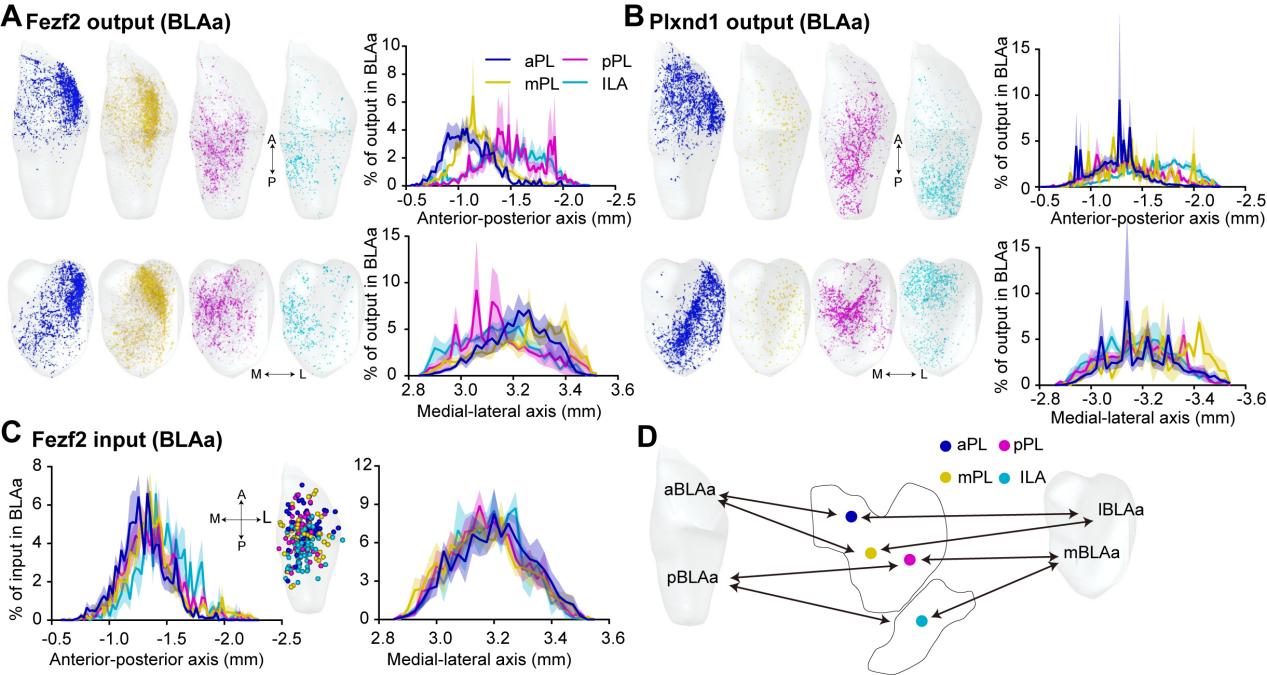


Figure S17: mPFC-BLAa connectivity. (A,B) Comparison of outputs in the BLAa of Fezf2 (A) and Plxnd1 (B) neurons in different mPFC subregions. Left panel, three-dimensional images of the axon fibers in the mPFC from representative samples. Right, density plot of outputs in the BLAa along the A-P axis or medial-lateral axis. (C) Left and right panels, density plot of inputs in the BLAa along the A-P axis or medial-lateral axis. Middle panel, three-dimensional representation of BLAa inputs to Fezf2 neurons in the mPFC subregions from representative samples. (D) Schematic of the connection patterns between the BLAa and mPFC subregions. a, anterior; p, posterior; m, medial; l, lateral.The density plot data in (A-C) are displayed as the average ± SEM, and the SEM is indicated by the shaded area. Data are from Fezf2 inputs: n = 5 (aPL), n = 7 (mPL and pPL), n = 3 (ILA); Fezf2 outputs: n =4 (aPL, mPL and ILA), n = 3 (pPL); Plxnd1 outputs: n = 5 (aPL), n = 3 (mPL, and ILA), n = 4 animals (pPL).SEM is indicated by the shaded area.


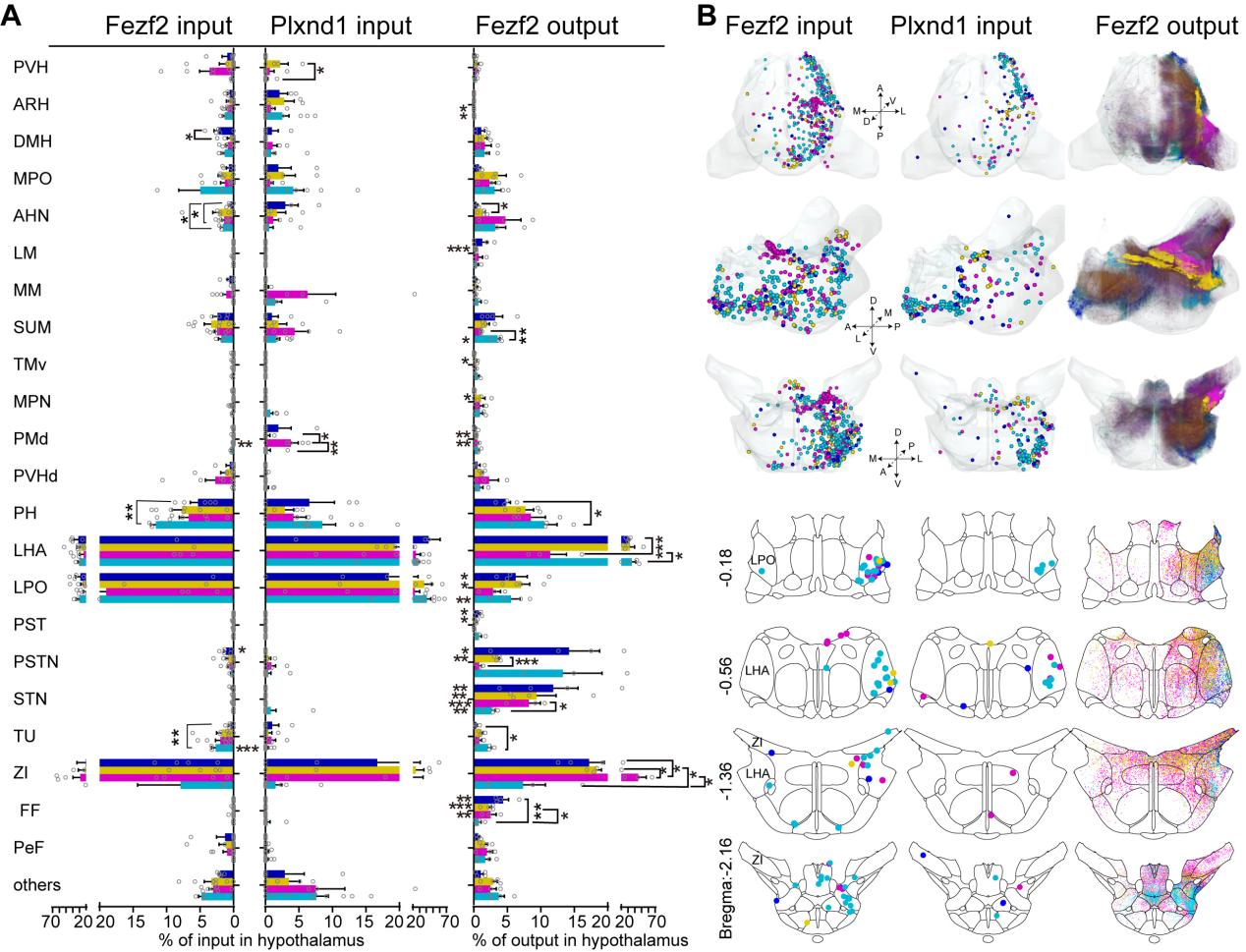


Figure S18: mPFC-hypothalamic connectivity. (A) Quantitative statistics of hypothalamic input and output of the mPFC subregions in Fezf2 and Plxnd1 neurons. The data are displayed as the average ±SEM, and data from Fezf2 inputs: n = 5 (aPL), n = 7 (mPL and pPL), n = 3 (ILA); Plxnd1 inputs: n = 4 (aPL and mPL), n = 5 (pPL), n = 9 (ILA); Fezf2 outputs: n =4 (aPL, mPL and ILA), n = 3 animals (pPL). A two-sided Student’s t-test was used to generate P values. Significant differences were labeled as ***p<0.001, **p<0.01, and *p<0.05. (B) Three-dimensional illustration and coronal sections (thickness: 100 μm) depicting input neurons to Fezf2 and Plxnd1 neurons or output fibers from Fezf2 neurons in the hypothalamus from representative samples. One dot represents one input neuron, while different colors reflect inputs to or outputs from different mPFC subregions. For detailed abbreviations, see Additional file 1: Table S1.


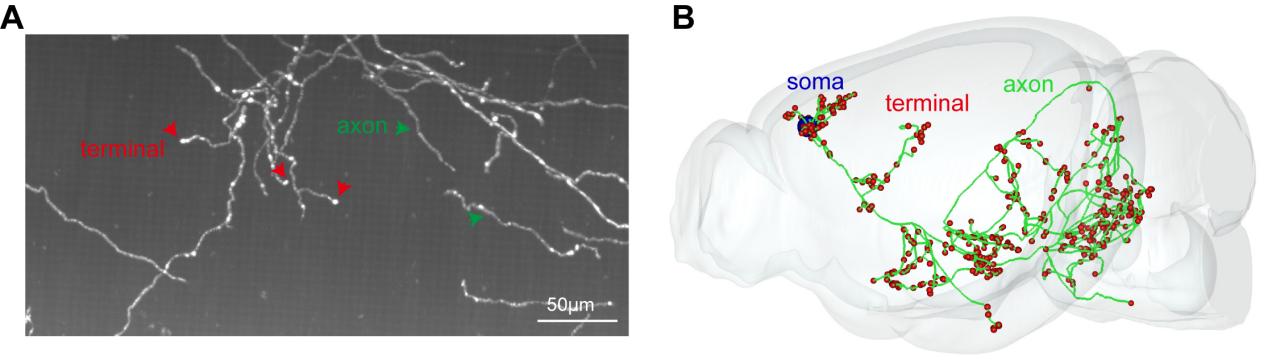


Figure S19: The axon terminals of the reconstructed single neurons. (A) Red arrowheads indicate the axon terminals, which are terminal boutons at the end of axon segments. (B) Single neuron morphology of PT neurons in the mPFC. Blue indicates the soma, green indicates the axon or dendrite fibers, and red indicates the axon or dendrite terminals.


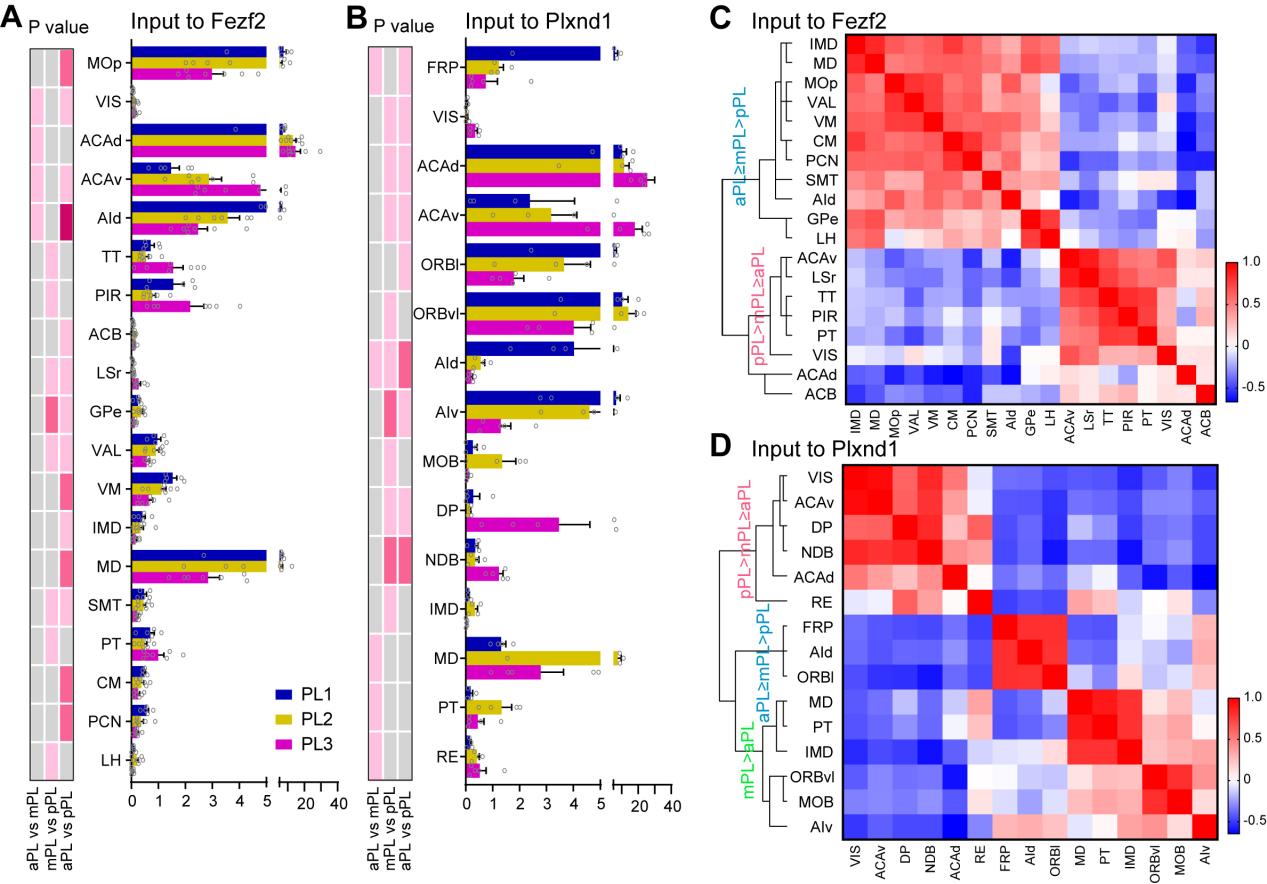


Figure S20: Brain regions with significant differences in the proportion of input between PL subregions. (A,B) Right panel, brain regions that differentially innervated Fezf2 (A) or Plxnd1 (B) neurons in the mPFC subregions. Left panel, p value heatmap between the PL subregions. A two-sided Student’s t-test was used to generate P values. The data are shown as the mean ± SEM, and the data are from Fezf2 inputs: n = 5 (aPL), n = 7 (mPL and pPL), n = 3 (ILA); Plxnd1 inputs: n = 4 (aPL and mPL), n = 5 (pPL), n = 9 animals (ILA). (C,D) Spearman correlation matrix and hierarchal clustering investigating the clustering of Fezf2 (C) and Plxnd1 (D) input brain regions with proportional differences based on their proportion of input to the PL subregions displayed in (A-B). Red, positive correlation; blue, negative correlation. For detailed abbreviations, see Additional file 1: Table S1.


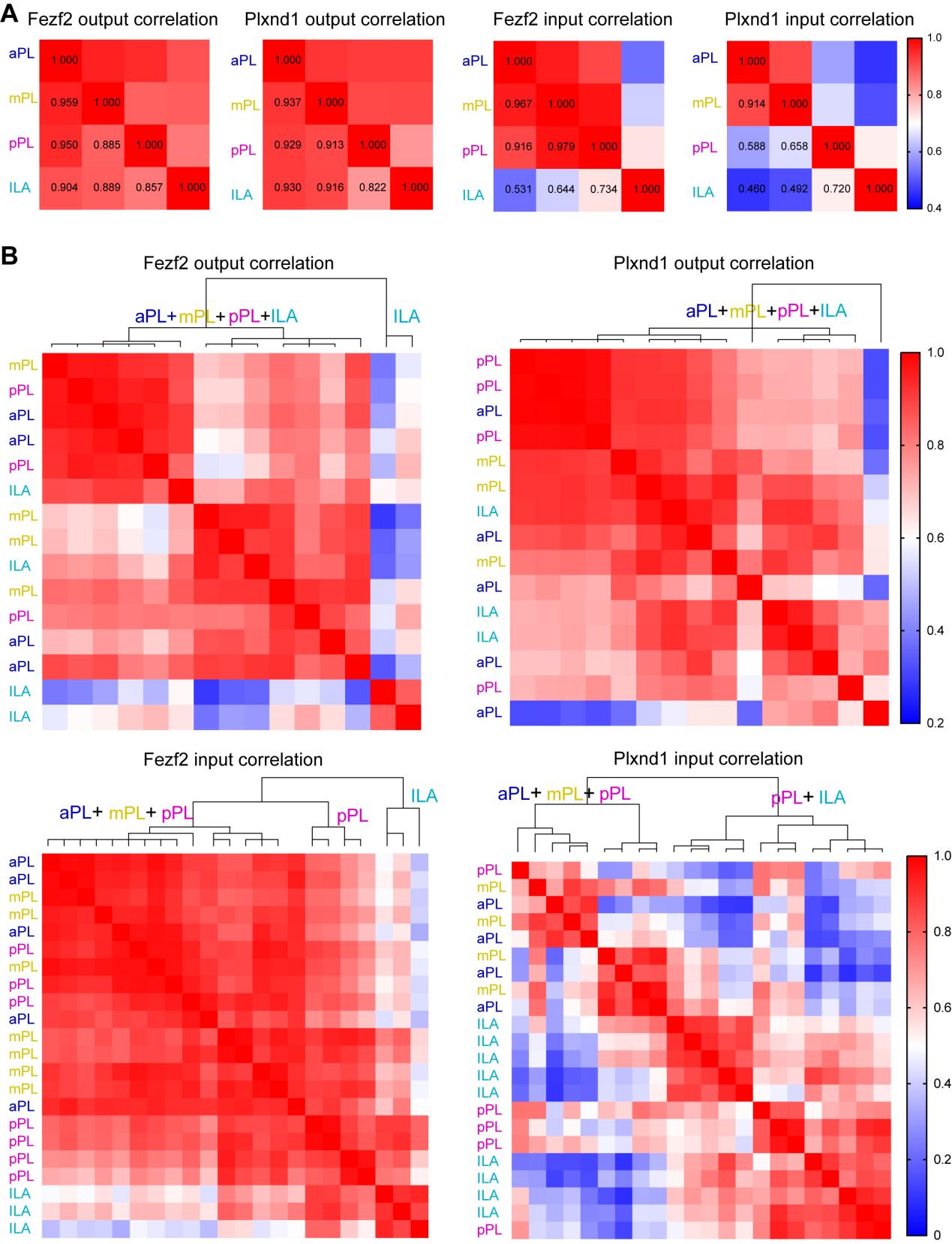


Figure S21: The correlation between the mPFC subregions according to their input-output connections. (A) Correlation coefficients between the four mPFC subregions according to their input and output connections. (B) Correlation and hierarchical cluster analysis of the individual input and output sample. The pairwise Pearson correlation coefficients were calculated according to the proportions of the 83 input brain regions, 116 Fezf2 output brain regions, and 53 Plxnd1 output brain regions displayed in Additional file 1: Fig. S7, 9, 10.

Table 1: More detailed abbreviations list.

| 3D: | Three-dimensional |
| --- | --- |
| AAA: | Anterior amygdalar area |
| ACA: | Anterior cingulate cortex |
| ACAd: | Anterior cingulate area, dorsal part |
| ACAv: | Anterior cingulate area, ventral part |
| ACB: | Nucleus accumbens |
| AD: | Anterodorsal nucleus of the thalamus |
| AHN: | Anterior hypothalamic nucleus |
| AId: | Agranular insular area, dorsal part |
| AIp: | Agranular insular area, posterior part |
| AIV: | Agranular insular cortex, ventral part |
| AIv: | Agranular insular area, ventral part |
| AM: | Anteromedial nucleus of the thalamus |
| AMd: | Anteromedial nucleus, dorsal part |
| AMv: | Anteromedial nucleus, ventral part |
| AOBgl: | Accessory olfactory bulb, glomerular layer |
| AON: | Anterior olfactory nucleus |
| A-P: | Anterior-posterior |
| aPL: | Prelimbic area, anterior part |
| APN: | Anterior pretectal nucleus |
| APr: | Area prostriata |
| ARA: | Allen Reference Atlas |
| ARH: | Arcuate hypothalamic nucleus |
| ATN: | Anterior thalamic nuclei |
| AUD: | Auditory areas |
| AV: | Anteroventral nucleus of the thalamus |
| BA: | Bed nucleus of the accessory olfactory tract |
| BLA: | Basolateral amygdalar nucleus |
| BLAa: | Basolateral amygdalar nucleus, anterior part |
| BLAp: | Basolateral amygdalar nucleus, posterior part |
| BLAv: | Basolateral amygdalar nucleus, ventral part |
| BMA: | Basomedial amygdalar nucleus |
| BMAa: | Basomedial amygdalar nucleus, anterior part |
| BMAp: | Basomedial amygdalar nucleus, posterior part |
| BST: | Bed nuclei of the stria terminalis |
| CA: | Ammon’s horn |
| CA1: | Field CA1 |
| CA1d: | Dorsal of CA1 |
| CA1i: | Intermediate of CA1 |
| CA1v: | Ventral of CA1 |
| CA2: | Field CA2 |
| CA3: | Field CA3 |
| CEA: | Central amygdalar nucleus |
| CEAc: | Central amygdalar nucleus, capsular part |
| CEAm: | Central amygdalar nucleus, medial part |
| CL: | Central lateral nucleus of the thalamus |
| CLA: | Claustrum |
| CM: | Central medial nucleus of the thalamus |
| COA: | Cortical amygdala area |
| COAa: | Cortical amygdalar area, anterior part |
| COAp: | Cortical amygdalar area, posterior part |
| CP: | Caudoputamen |
| CS: | Superior central nucleus raphé |
| CT: | Corticothalamic |
| CUN: | Cuneiform nucleus |
| DG: | Dentate gyrus |
| DMH: | Dorsomedial nucleus of the hypothalamus |
| DP: | Dorsal peduncular area |
| DR: | Dorsal nucleus raphe |
| ECT: | Ectorhinal area |
| ENTl: | Entorhinal area, lateral part |
| ENTm: | Entorhinal area, medial part |
| EPd: | Endopiriform nucleus, Dorsal part |
| EPv: | Endopiriform nucleus, Ventral part |
| FF: | Fields of forel |
| fMOST: | Fluorescence micro-optical sectioning tomography |
| FRP: | Frontal pole |
| FS: | Fundus of striatum |
| GMA: | Glycol methacrylate |
| GPe: | Globus Pallidus, External segment |
| GPi: | Globus Pallidus, Internal segment |
| GRN: | Gigantocellular reticular nucleus |
| GU: | Gustatory areas |
| HATA: | Hippocampal-amgdalar transition area |
| HPF: | Hippocampal formation |
| IA: | Intercalated amygdalar nucleus |
| IAD: | Interanterodorsal nucleus of the thalamus |
| IAM: | Interanteromedial nucleus of the thalamus |
| IG: | Induseum griseum |
| ILA: | Infralimbic area |
| ILM: | Intralaminar thalamic nuclei |
| IMD: | Intermediodorsal nucleus of the thalamus |
| IO: | Inferior olivary complex |
| IPL: | Interpeduncular nucleus, lateral |
| IRN: | Intermediate reticular nucleus |
| IT: | Intratelencephalic |
| LA: | Lateral amygdalar nucleus |
| LD: | Lateral dorsal nucleus of the thalamus |
| LDT: | Laterodorsal tegmental nucleus |
| LH: | Lateral habenula |
| LHA: | Lateral hypothalamic area |
| LM: | Lateral posterior nucleus of the mammillary nucleus |
| LP: | Lateral posterior nucleus of the thalamus |
| LPO: | Lateral preoptic area |
| LSr: | Lateral septal nucleus, rostral |
| MA: | Magnocellular nucleus |
| MARN: | Magnocellular reticular nucleus |
| MD: | Mediodorsal nucleus of the thalamus |
| MDRNd: | Medullary reticular nucleus, dorsal part |
| MEA: | Medial amygdalar nucleus |
| MED: | Medial dorsal thalamic nuclei |
| MM: | Medial mammillary nucleus |
| MOB: | Main olfactory bulb |
| MOp: | Primary motor area |
| MOs: | Secondary motor area |
| mPFC: | Medial prefrontal cortex |
| mPL: | Prelimbic area, middle part |
| MPN: | Medial preoptic nucleus |
| MPO: | Medial preoptic area |
| MRN: | Midbrain reticular nucleus |
| MS: | Medial septal nucleus |
| MT: | Medial terminal nucleus of the accessory optic tract |
| MTN: | Midline thalamic nuclei |
| NDB: | Diagonal band nucleus |
| NLOT1: | Nucleus of the lateral olfactory tract, molecular layer |
| NLOT3: | Nucleus of the lateral olfactory tract, layer 3 |
| NPC: | Nucleus of the posterior commissure |
| NTS: | Nucleus of the solitary tract |
| ORB: | Orbital area |
| ORBl: | Orbital area, lateral part |
| ORBm: | Orbital area, medial part |
| ORBvl: | Orbital area, ventrolateral part |
| OT: | Olfactory tubercle |
| PA: | Posterior amygdalar nucleus |
| PAA: | Piriform-amygdalar area |
| PAG: | Periaqueductal gray |
| PAR: | Parasubiculum |
| PARN: | Parvicellular reticular nucleus |
| PB: | Parabrachial nucleus |
| PBS: | Phosphate buffered solution |
| PCG: | Pontine central gray |
| PCN: | Paracentral nucleus of the thalamus |
| PeF: | Perifornical nucleus |
| PERI: | Perirhinal area |
| PF: | Parafascicular nucleus |
| PFA: | Paraformaldehyde |
| PG: | Pontine gray |
| PH: | Posterior hypothalamic nucleus |
| PIR: | Piriform area |
| PL: | Prelimbic area |
| PMd: | Dorsal premammillary nucleus |
| PO: | Posterior complex of the thalamus |
| POST: | Postsubiculum |
| pPL: | Prelimbic area, posterior part |
| PPN: | Pedunculopontine nucleus |
| PR: | Perireunensis nucleus |
| PRC: | Precommissural nucleus |
| PRE: | Presubiculum |
| PRNc: | Pontine reticular nucleus, caudal part |
| PRNr: | Pontine reticular nucleus |
| Pros: | Prosubiculum |
| PST: | Preparasubthalamic nucleus |
| PSTN: | Parasubthalamic nucleus |
| PT: | Parataenial nucleus |
| PVH: | Paraventricular hypothalamic nucleus |
| PVHd: | Paraventricular hypothalamic nucleus, descending division |
| PVT: | Paraventricular thalamic nucleus |
| RE: | Nucleus of reunions |
| RH: | Rhomboid nucleus |
| RM: | Nucleus raphé magnus |
| RN: | Red nucleus |
| RPA: | Nucleus raphe pallidus |
| RPO: | Nucleus raphe pontis |
| RR: | retrorubral area |
| RSP: | Retrosplenial area |
| RSPagl: | Retrosplenial area, lateral agranular part |
| RSPd: | Retrosplenial area, dorsal part |
| RSPv: | Retrosplenial area, ventral part |
| RT: | Reticular nucleus of the thalamus |
| SCdg: | Superior colliculus, motor related, deep gray layer |
| SCdw: | Superior colliculus, motor related, deep white layer |
| SCig: | Superior colliculus, motor related, intermediate gray |
| SCiw: | Superior colliculus, motor related, intermediate white layer |
| SH: | Septohippocampal nucelus |
| SI: | Substantia innominata |
| SMT: | Submedial nucleus of the thalamus |
| SNc: | Substantia nigra, compact part |
| SNr: | Substantia nigra, reticular part |
| SPA: | Subparafascicular area |
| SPF: | Subparafascicular nucleus |
| SPVC: | Spinal nucleus of the trigeminal, caudal part |
| SSp: | Primary somatosensory area |
| SSs: | Supplemental somatosensory area |
| STN: | Subthalamic nucleus |
| SUB: | Subiculum |
| SUM: | Supramammillary nucleus |
| TEa: | Temporal association areas |
| TM: | Tamoxifen |
| TMv: | Tuberomammillary nucleus, ventral part |
| TR: | Postpiriform transition area |
| TRN: | Tegmental reticular nucleus |
| TT: | Taenia tecta |
| TTd: | Taenia tecta, dorsal part |
| TTv: | Taenia tecta, ventral part |
| TU: | Tuberal nucleus |
| VAL: | Ventral anterior-lateral complex of the thalamus |
| VENT: | Ventral thalamic nuclei |
| VIS: | Visual areas |
| VISC: | Visceral area |
| VM: | Ventral medial nucleus of the thalamus |
| VPL: | ventral posterolateral nucleus of the thalamus |
| VPM: | Ventral posteromedial nucleus of the thalamus |
| VTA: | Ventral tegmental area |
| Xi: | Xiphoid nucleus |
| ZI: | Zona incerta |
